# The Prediction of miRNAs in SARS-CoV-2 Genomes: hsa-miR Databases Identify 7 Key miRs Linked to Host Responses and Virus Pathogenicity-Related KEGG Pathways Significant for Comorbidities

**DOI:** 10.3390/v12060614

**Published:** 2020-06-04

**Authors:** Elif Damla Arisan, Alwyn Dart, Guy H. Grant, Serdar Arisan, Songul Cuhadaroglu, Sigrun Lange, Pinar Uysal-Onganer

**Affiliations:** 1 Institute of Biotechnology, Gebze Technical University, Gebze, 41400 Kocaeli, Turkey; d.arisan@gtu.edu.tr; 2 Institute of Medical and Biomedical Education, St George’s University of London, Cranmer Terrace, Tooting, London SW17 0RE, UK; ddart@sgul.ac.uk; 3 School of Life Sciences, University of Bedfordshire, Park Square, Luton LU1 3JU, UK; guy.grant@beds.ac.uk; 4 Department of Urology, Şişli Hamidiye Etfal Research and Training Hospital, 34360 Istanbul, Turkey; serdararisan@gmail.com; 5 Thoracic Surgery Clinic, Memorial Hospital Sisli, Kaptanpasa Mah. Piyalepasa Bulvarı, 434385 Istanbul, Turkey; cuhadaroglusongul@hotmail.com; 6 Tissue Architecture and Regeneration Research Group, School of Life Sciences, University of Westminster, London W1W 6UW, UK; s.lange@westminster.ac.uk; 7 Cancer Research Group, School of Life Sciences, University of Westminster, London W1W 6UW, UK

**Keywords:** COVID-19, SARS–CoV-2, coronavirus, microRNAs (miRs 8066, 5197, 3611, 3934-3p, 1307-3p, 3691-3p, 1468-5p), viral pathogenesis, cell signalling pathways, comorbidities

## Abstract

Severe acute respiratory syndrome coronavirus-2 (SARS-CoV-2) is a member of the *betacoronavirus* family, which causes COVID-19 disease. SARS-CoV-2 pathogenicity in humans leads to increased mortality rates due to alterations of significant pathways, including some resulting in exacerbated inflammatory responses linked to the “cytokine storm” and extensive lung pathology, as well as being linked to a number of comorbidities. Our current study compared five SARS-CoV-2 sequences from different geographical regions to those from SARS, MERS and two cold viruses, OC43 and 229E, to identify the presence of miR-like sequences. We identified seven key miRs, which highlight considerable differences between the SARS-CoV-2 sequences, compared with the other viruses. The level of conservation between the five SARS-CoV-2 sequences was identical but poor compared with the other sequences, with SARS showing the highest degree of conservation. This decrease in similarity could result in reduced levels of transcriptional control, as well as a change in the physiological effect of the virus and associated host-pathogen responses. MERS and the milder symptom viruses showed greater differences and even significant sequence gaps. This divergence away from the SARS-CoV-2 sequences broadly mirrors the phylogenetic relationships obtained from the whole-genome alignments. Therefore, patterns of mutation, occurring during sequence divergence from the longer established human viruses to the more recent ones, may have led to the emergence of sequence motifs that can be related directly to the pathogenicity of SARS-CoV-2. Importantly, we identified 7 key-microRNAs (miRs 8066, 5197, 3611, 3934-3p, 1307-3p, 3691-3p, 1468-5p) with significant links to KEGG pathways linked to viral pathogenicity and host responses. According to Bioproject data (PRJNA615032), SARS-CoV-2 mediated transcriptomic alterations were similar to the target pathways of the selected 7 miRs identified in our study. This mechanism could have considerable significance in determining the symptom spectrum of future potential pandemics. KEGG pathway analysis revealed a number of critical pathways linked to the seven identified miRs that may provide insight into the interplay between the virus and comorbidities. Based on our reported findings, miRNAs may constitute potential and effective therapeutic approaches in COVID-19 and its pathological consequences.

## 1. Introduction

The emergence of SARS-coronavirus-2 (SARS-CoV-2), which causes coronavirus-mediated disease 2019, or COVID-19, has now spread pandemically, resulting in a serious global health crisis. Coronaviruses are positive-single stranded RNA (+ssRNA) zoonotic viruses with a ~30 kb genome (approx). The coronavirus subfamily is divided into four genera: α, β, γ, and δ, based on serotype and genome features. The genome of a typical CoV codes for at least 6 different open reading frames (ORFs), which have variations based on the CoV type 4. Some ORFs encode non-structural proteins while others code for structural proteins required for viral replication and pathogenesis. Structural proteins include the spike (S) glycoprotein, which has various roles in SARS-CoV based on sequence analysis and might share similar viral genomic and transcriptomic complexity. Other proteins include matrix (M) protein, small envelope (E) protein, and nucleocapsid (N) protein for virus entrance and spread [1]. SARS-CoV-2 infections mainly target the lungs with respect to other viral infections, which begin with upper respiratory tract symptoms. However, it is obvious that an important differentiating feature of the current SARS-CoV-2 infection is that it does not follow regular viral lower respiratory infection pathways. There are a wide variety of clinical symptoms presented by SARS-CoV-2 infection. Mainly (otherwise unexplained) fever and a failure to breathe fully are the most frequent symptoms, with non-productive cough, sneezing, sudden loss of smell and/or taste, pain in the chest cavity (mostly anteriorly), headache and prominent muscle ache, abdominal pain with diarrhoea, and neurological symptoms with facial nerve involvement [2]. Patients are generally referred to hospital when fever does not subside for 2 to 3 days, or respiratory effort gets harder. Clinical observation indicates that elderly patients present more unexplained fever for 3 days, in contrast to younger patients who present with difficulty in breathing without fever. In addition, the current lack of successful therapeutic intervention strategies to prevent the disease, or overcome serious mortality rates as a result of difficult lung pathophysiology mediated by SARS-CoV-2 infection, requires clarification of the molecular aetiology behind the mild or severe conditions of the disease. Increasing mortality rates are the major obstacle in the management of the disease in existing healthcare systems, which have insufficient numbers of intensive care units. Clinically, the initiation of acute respiratory distress syndrome (ARDS), which is the predominant severe pathology, leads to diffuse alveolar damage (DAD). In addition, the presence of a cytokine storm, the excessive and uncontrolled release of pro-inflammatory cytokines such as IL-6, TNFα, IL-1β, IL-8, and IL2R, is associated with ARDS, hypercoagulation and increased erythematosus sedimentation rate (ESR) [3]. SARS-CoV-2, as well as other viruses, leads to pathophysiological problems in the host cells, and alters the expression of a number of genes. Thus, clarification of molecular regulators on human cells is important to evaluate molecular mechanisms.

MicroRNAs (miRs) are non-coding RNAs of length approximately 20–22 nucleotides; they post-transcriptionally regulate gene expression by binding to the 3′-untranslated regions of target mRNAs, leading to degradation or translational inhibition. Each miR can target hundreds of mRNAs within a given cell type, and a single mRNA is often the target of multiple miRs, and thus over half of the human transcriptome is predicted to be under miR regulation, embedding this post-transcriptional control pathway within nearly every biological process [4,5]. Virally expressed miRs have recently been discovered, especially in viruses with DNA genomes. The best-known viral miRs are found mostly in herpesvirus families, where they enhance bilateral crosstalk between viral pathogenesis and host response mechanisms. Additionally, it has been shown that different virus families such as delta bovine leukaemia virus and foamy retroviruses could encode miRs [6]. As previously shown, viral miRs are critical in the immune evasion mechanisms, affecting host immunity-related gene regulation networks. This bilateral effect results in a rapid increase during the virus resistance against host defence mechanisms and leads to their survival in host cells [7]. The scope of miR generation from ssRNA viral genomes has been controversial, mainly due to the potentially deleterious effect of ssRNA viral genome cleavage into pre-/pri-miRs, making it unavailable for packaging into new virus particles. However, predictive studies on RNA viral genomes reveal RNA structures, which are conceivable Drosha and Dicer substrates. One of the well-described annotations was performed for HIV to define HIV-1 TAR RNA, a 59-nt long sequence that could generate a stem-loop structure similar to the pre-miR structure [8]. The identified numbers of viral miRs are not as many as for other organisms. One of the reasons for this is that viral miRs, due to their cytoplasmic location in host cells, render interaction with nuclear miR biogenesis elements of the host cells. Another hypothesis is that the cleavage of the viral RNA genome could generate miRs [9]. According to data obtained using *in silico* screening, there are fewer identified virus miRs compared with other species, and their functional roles in host cells are not well described. Therefore, greater effort is required to identify novel virally encoded miRs and to predict their host targets. Conversely, host cell miRs may alter the biological effect of the viruses [10]. Several interactions between viruses and the miRs in the host cells have been reported: the virus may either avoid being targeted by the cellular miRs [11]; block the cellular miRs to regulate key proteins in main signalling pathways [12,13]; synthesize their own viral miRs to create a more favourable cellular environment to survive in the host cells [14], or simply use the cellular miRs to their own advantage [15]. It should also be noted that host cell miR repertoires change dramatically in response to various diseases. Several underlying cardiovascular and lung conditions may significantly alter host miR expression, which would affect virus-host lung cell interactions, and may significantly determine the course of the disease.

The existence of sequences within the ssRNA viral genome with a high degree of sequence similarity to human (or mammalian) sequences is unlikely to be accidental. Hypothetically, some viral ssRNA molecules may be channelled into the miR processing pathways influencing the host cell, whilst other ssRNAs are packaged, combinations of which may increase overall viral activity. The general prediction mechanism of putative miRs is based on the determination of seed region specificity. The seed sequence, which is the critical part of the target prediction, is essential for the binding of the miR to the target mRNA. The seed sequence or seed region is referred to as an evolutionary conserved heptameric sequence, which is mostly situated at positions 2–7 from the miR 5’-end. Therefore, point mutations at seed regions are critical to evaluate the target specificity and functional consequences of the potential miR and target mRNA. The complex interaction between the viruses and the host miRs mostly become more advantageous for the viruses as they enable them to avoid the immune system response and allow them to employ the host’s miRs [16]. Recently it has been suggested that miRs play a role in the host’s defence system against viral infections such as HIV-1, HSV, HCV, dengue and influenza [17,18,19,20,21]. Therefore, targeting specific miRs could prove to be a novel strategy for treatment. The best-known anti-miR treatment for viral infection is targeting miR-122 to cure HCV [18]. It has also been reported that anti-miR-based HCV therapy is genotype independent, which makes it safe, effective and well tolerated by patients [22].

In this study, our first aim was to identify human miRs that show sequence similarities to the SARS-CoV-2 genome, and their conservation ratio in SARS-CoV-2 isolates obtained from different geographical regions. Following determination of significantly similar miR sequences, we evaluated their potential effect on host cells through analysis of their target genes and related KEGG and GO pathways using bioinformatics tools. In the final part of the study, the miR-mediated alterations of different pathways were compared to public transcriptome data obtained from SARS-CoV-2-infected cell and tissue biopsy samples. To this end, the study aims to clarify the role of potential miR-mimic sequences in the SARS-CoV-2 genome with their host target genes, which may propose a new perspective for antiviral strategies.

## 2. Material and Methods

### 2.1. Genome Sequences

The SARS-CoV-2 genome sequences from China, Italy, Spain (Valencia), and those for MERS, SARS, OC43 and 229E were obtained from NCBI (GenBank: NC_045512.2, LC528232.1, MT066156.1, MT198652.2, KT225476.2, NC_004718.3, NC_006213.1, NC_002645.1, respectively). The SARS-CoV-2 genome sequences from England (hCoV-19/England/20136087804/2020|EPI_ISL_420910, no treatment) and Turkey (hCoV-19/Turkey/GLAB-CoV008/2020) were obtained from the China National Bioinformatics Center, GISAID database [23] (https://www.gisaid.org). In addition, a SARS-CoV-2 strain isolated from a Turkish patient, and infected to Vero E6 cells passage 4 sequence (hCoV-19/Turkey/ERAGEM-001/2020;) was used for alignment studies with the miRBase mature miRNA search tool.

### 2.2. miR Prediction 

The miRTarget and miRBase programmes were used to predict the similarities between the SARS-CoV-2 genome and human miRs; e-value <10 and score >70 were considered as significant. DianaTools miRPath V3 were then used to create heat maps for pathways affected by selected miRs, focusing on the microT-CDS version 5.0 database. The *p* value threshold was 0.05 and microT threshold was 0.8. Heatmap analysis was done with pathway intersection [24].

### 2.3. Mutational Analysis of Potential miRNA Sites

Viral genome sequencing data was obtained from the GISAID database (https://www.gisaid.org), and analysed as multiple sequence alignments using the Clustal Omega at EBI (www.ebi.ac.uk/Tools/msa/clustalo/).

### 2.4. Pathway Analysis

Bioproject data was obtained from PRJNA615032 bioproject trancriptome data, which includes lung biopsies from SARS-CoV-2-infected patients and healthy volunteers as well as mock and SARS-CoV-2-transfected NHEB and A549 cell lines. The data have been deposited with links to BioProject accession number PRJNA615032 in the NCBI BioProject database (https://www.ncbi.nlm.nih.gov/bioproject/). All the selected data were reanalysed at the Rosalind bioinformatics server. Data analysis was performed according to 1.5 fold change between untransfected and transfected cell lines in a data pool calculation for both cell lines at *p* < 0.05 significance level. Data was analyzed by Rosalind (https://rosalind.onramp.bio/), with a HyperScale architecture developed by OnRamp BioInformatics, Inc. (San Diego, CA, USA). Reads were trimmed using cutadapt [25]. Quality scores were assessed using FastQC [26]. Reads were aligned to the *Homo sapiens* genome built by GRCh38 using STAR [27]. Individual sample reads were quantified using HTseq [28] and normalized via relative log expression (RLE) using DESeq2 R library [29]. Read distribution percentages, violin plots, identity heatmaps, and sample MDS plots were generated as part of the QC step using RSeQC [30]. DEseq2 was also used to calculate fold changes and *p*-values and perform optional covariate correction. Clustering of genes for the final heatmap of differentially expressed genes was done using the PAM (partitioning around medoids) method using the fpc R library (https://cran.r-project.org/web/packages/fpc/index.html). Hypergeometric distribution was used to analyze the enrichment of pathways, gene ontology, domain structure, and other ontologies. The topGO R library [31], was used to determine local similarities and dependencies between GO terms in order to perform Elim pruning correction. Several database sources were referenced for enrichment analysis, including Interpro [32], NCBI [33], MSigDB [34,35], REACTOME [36], and WikiPathways [37]. Enrichment was calculated relative to a set of background genes relevant for the experiment.

## 3. Results

### 3.1. Analysis of SARS-CoV-2 Viral Genome for miR Sequences with High Human Similarity and Functional Characterisation

The miRBase online database holds 2565 miR sequences and from these we identified regions of the SARS-CoV-2 viral genome, which showed high similarity to human miRs. Similarly, we analysed SARS-CoV-2 genomes obtained from different geographical areas for comparison. Despite the relatively large SARS-COV-2 genome, only a few miRs were found to show similarities with human miRs (Table 1).

**Figure 1:**
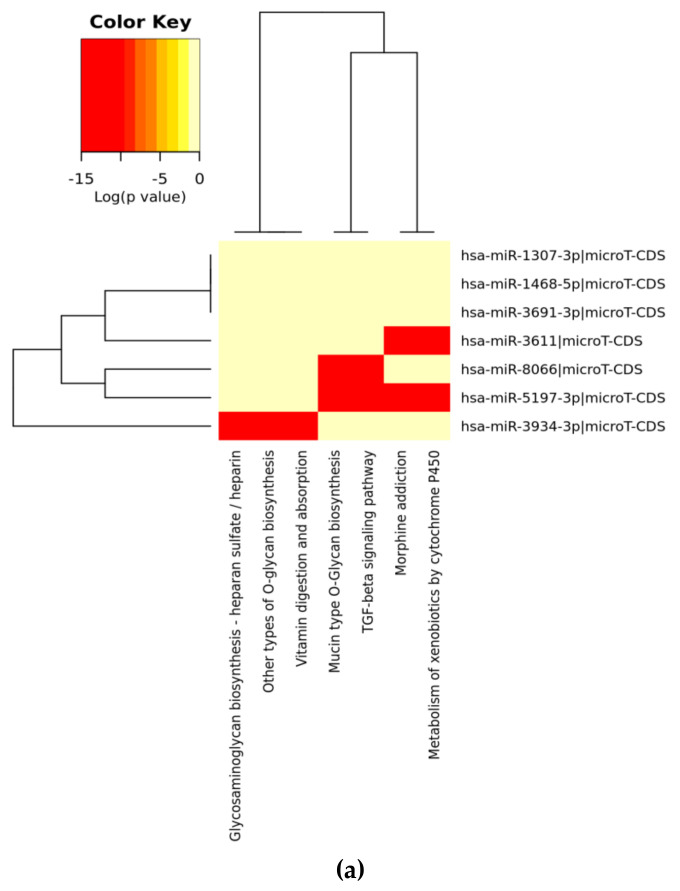

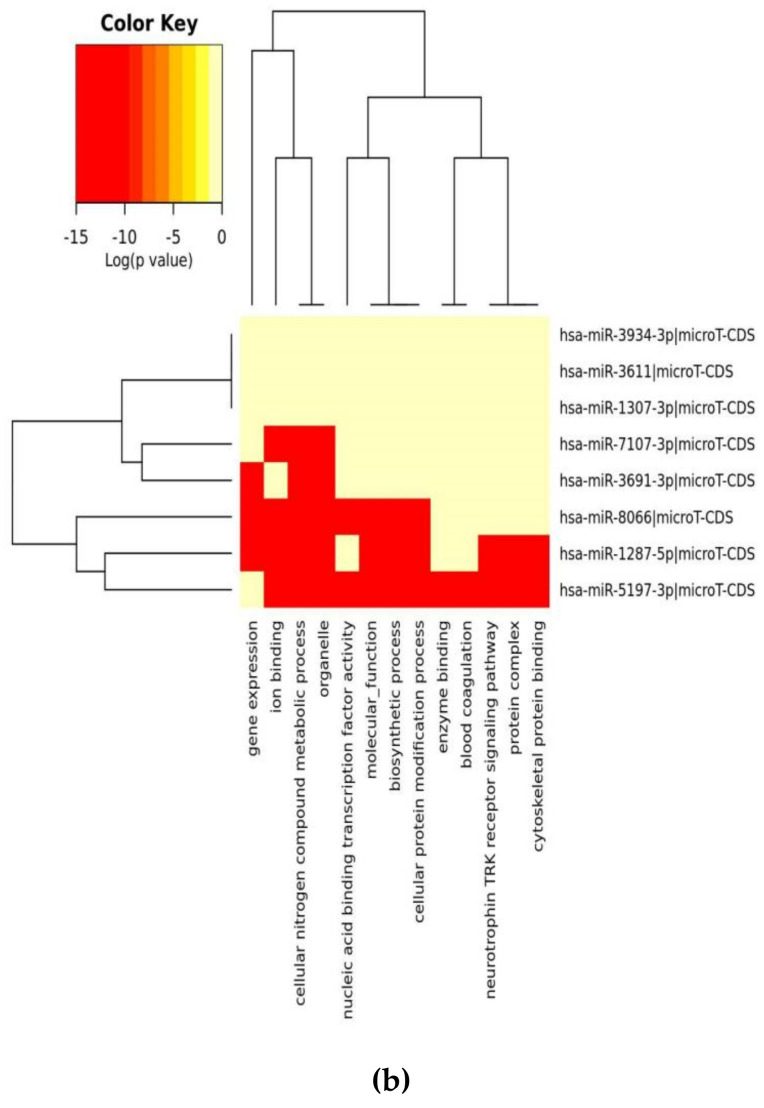
Heat map analysis of KEGG pathway (**A**) and GO analysis (**B**) for selected miRs on the microT-CDS database. The heat map is drawn with miRPATH (version 3). Neighbourhood lines indicate the shared target mRNAs found in a defined pathway.

We have found five highly significant miRs from four different countries; Turkey, Italy, Spain, and the UK; one RefSeq sequence from Wuhan and one SARS-CoV-2 genome from the VeroE6 cell line: **miR-8066** (e-values; 1.6 for Wuhan, 2.8 for Valencia, 1.6 for both Italy and England, 2.8 for VeroE6 cells SARS-CoV-2 genomes); **miR-5197-3p** (e-values; 1.6 for Wuhan, 2.1 for VeroE6 cells, 2.8 for Valencia and 1.9 for both Italy and England SARS-CoV-2 genomes), and **miR-3611** (e-values; 2.8 for Wuhan, 3.3 for Valencia and 2.8 for both Italy and England, 2.8 for VeroE6 cells SARS-CoV-2 genomes); **miR-3934-3p** (e-values; 3.4–5.0), and **miR-1307-3p** (e-values; 4.3–6.3). We could, however, detect a similar alignment with **miR-1307-3p** in SARS-CoV-2-infected Vero E6 cells. Additionally, we found that the same miR sequences exist within four genomes of SARS-CoV-2, within the lower e-values of 5.0–10.0 were **miR-3691-3p** and **miR-1468-5p**. Again, **miR-3691-3p** was not a positive hit in the SARS-CoV-2-infected Vero E6 cell line. All of these miR similarities to human miRs were conserved in all studied genomes. We then used DianaTools to identify the potential pathways to which these miRs contribute (Figure 1). The functional characterizations of these highly conserved miRs were analyzed with KEGG molecular pathways. The selected intersected pathways were analyzed as significant targets as *p* value 0.05 with threshold value 0.8 and Fisher’s Exact Test (hypergeometric distribution) calculations by miRPath version 3.0 in the microT-CDS database. As shown in Figure 1. **miR-8066** and **miR-5197-3p** are critical on TGF-β and mucin type O-Glycan biosynthesis pathways. **miR-8066** is also related to cytokine-cytokine receptor interaction. **miR-5197** is significantly related to morphine addiction and metabolism of xenobiotics by cytochrome P450 mechanisms. These two miRs were highly conserved, and their coexistence was significant within the four-genome search. **miR-3611** was the other leading miR, which possesses co-occurrence potential with **miR-8066 and miR-5197** in all genomes that were effective on GABAergic synapse, morphine addiction and metabolism of xenobiotics by cytochrome P450 mechanisms. Although co-occurrences of **miR-1468-5p and miR-1307-3p** were similar to **miR-3611**, it was less effective on all evaluated metabolic pathways. **miR-3934-3p** was effective on glycosaminoglycan biosynthesis—heparan sulfate/heparin, other types of O-glycan biosynthesis, and vitamin digestion-absorption mechanisms, respectively.

When we evaluated significant union gene-based pathway alterations for selected miRNAs, we found that mucin type O-glycan biosynthesis, morphine addiction, TGF-β signaling pathway, axon guidance and GABAergic synapse mechanisms were significantly affected according to KEGG pathway analysis on the miRPath (Table 2A).

In a similar setting, GO enrichment analysis results were again obtained from miRPath analysis. We determined that the clusters, based on the selected miRs’ target genes, were organelle, cellular nitrogen compound metabolic process, ion binding, biosynthetic process, nucleic acid binding transcriptional factor activity, and cellular protein modification process (Table 2B).

In the current analysis, we detected mutations in **miR-1307** and **miR-8066** only. Additionally, a mutation on **miR-129-2-3p** was found on only the Icelandic SARS-CoV-2 genome (Table 3). Moreover, **miR-129-2-3p** is one of the selected miR (Table 1) found only in the Wuhan and Italy genomes, although in less than 5% of the genomes sequenced. These three miRs are potentially involved in mucin type O-glycan biosynthesis, TGF-β signalling pathway, amphetamine addiction, cytokine-cytokine receptor interaction and nicotinate-nicotinamide metabolism. All of these pathways are associated with host responses against SARS-CoV-2, and virus pathogenesis in host cells. The mutations on selected miRs may affect their presence in different strains and may alter their potential host-mediated responses. The remainder of the miRs, presented in Table 3, was found to be conserved. Therefore, our data suggest that either these sequences are crucial for SARS-CoV-2, or their locations are important for the virus to survive. Using the GISAID database, we analysed a sample of viral genomic sequences from several geographical areas for mutations in the potential miR sequences (*n* = 28–133). The majority of the miRs studied showed very few base changes in these sequences, with <1% overall. MiRs **miR-1468-5p** and, particularly, **miR-1307-3p** showed an increased percentage of mutations. All the mutations analysed reduced the microRNA base similarity and decreased the score value to below significance (<70).

Due to the increased mutation ratios in SARS-CoV-2 strains isolated from different geographical regions, we checked the conservation of miR mimic sequences in different sequence results. The comparison of depicted miR sequences from different SARS-Cov-2 strains (Table S1), with the Wuhan SARS-CoV-2 (HCOV-19/WUHAN/WH01/2019|EPI_ISL_406798|2019-12-26), was analyzed by BLAST nucleotide search with default values.

### 3.2. Analysis of Gene Alterations in NHEB Bronchial Epithelial and A549 Cells Due to SARS-CoV-2 Infection

In the final part of the study, we analyzed the gene expression alterations due to SARS-CoV-2-mediated infection in NHEB bronchial epithelial cells and A549 lung cancer cell lines. For this purpose, we used the Bioproject PRJNA615032 publicly available data with the Rosalind bioinformatics data analysis server.

**Figure 2:**
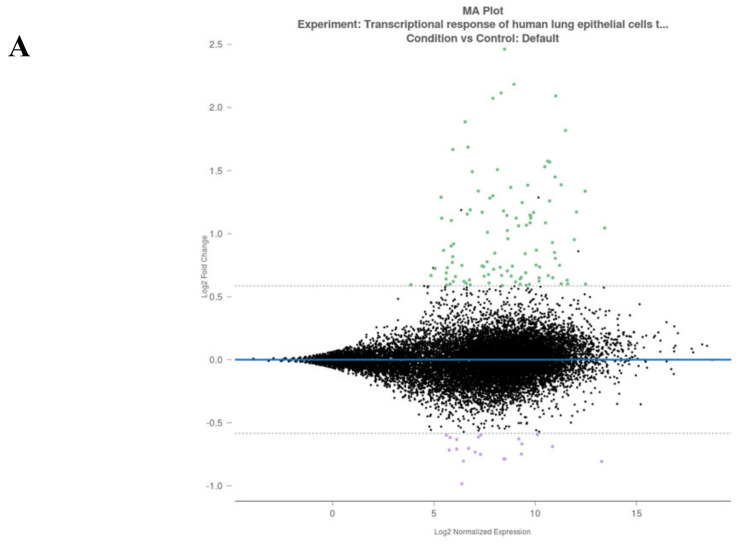

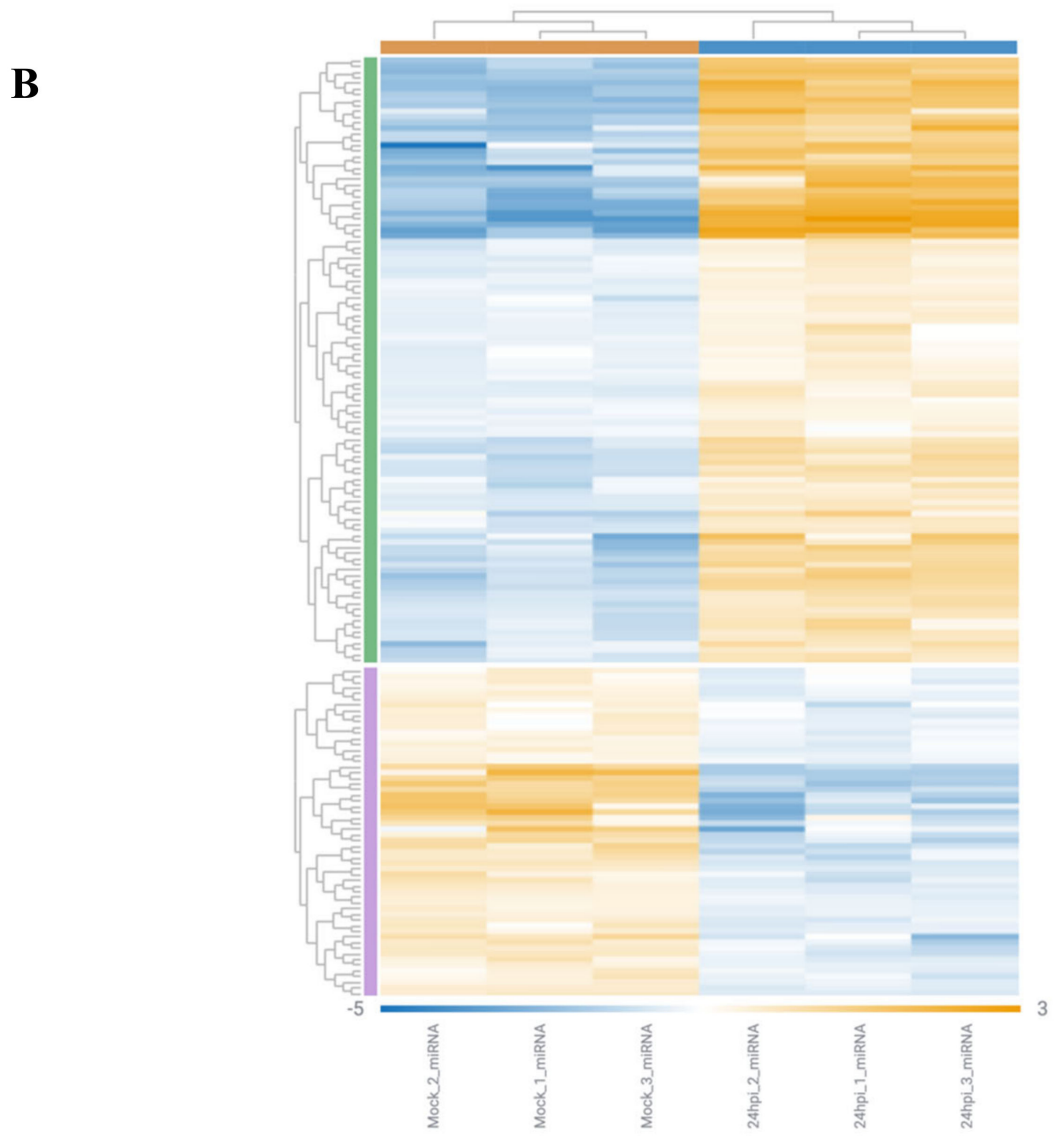
Rosalind meta-analysis for Bioproject PRJNA615032 was used for differential gene expression between SARS-CoV-2-infected NHEB and A549 cells with their (mock treated) controls (*n* = 3). 1.5 fold change was accepted as threshold value. (A) MA plot view of differential expression of upregulated and downregulated genes. (B) Heatmap analysis of each clone for 204 differentially expressed gene targets.

The meta-analysis results were evaluated for the differential gene expression at a 1.5 fold change cut-off level. In total, 124 genes were selected according to a statistical *p* value threshold of <0.05, and analyzed for the related signaling axis to understand the disease pathophysiology, as shown in Figure 2A,B. While 104 genes were upregulated in SARS-CoV-2-infected cells, 20 genes were downregulated.

All of these genes were also analysed using cluster analysis tools provided by the Rosalind bioinformatic data analysis server for different pathways. As shown in Table 4, the Wiki pathways, Bioplanet, KEGG, REACTOME, Panther, Pathway Interaction DB, and the number of virus-host response pathways, were significantly altered. These significantly altered pathways showed correspondingly similar patterns with SARS-CoV-2-mediated known clinical pathologies. These pathways were based on major differences of the target genes for inflammation responses.

The predicted pathways in cell lines showed similar significance with selected miRs that mainly target inflammation and virus pathogenesis (Figure 1 and Table 2). Thus, we concluded that the selected miRs, that showed high similarity with human miRs, are also the critical targets, which project the major clinical pathophysiological conditions related to pathway alterations.

## 4. Discussion

In the current study, we identified potentially similar miR sequences of human miRs and SARS-Cov-2 strains from different geographical regions. For this purpose, we selected different genome sequence studies released recently in PubMed® and GISAID databases, which included genome results for SARS-CoV-2 strains from four different geographical regions. We first aligned all the sequences with the mature hsa-miR database presented in miRBase. During this study, we made a cluster according to the co-existence of significantly aligned hsa-miRs with the SARS-CoV-2 genome and their biological significance in human cells. miRPath version 3 was used for the determination of the selected miRs’ potential biological effects via searching target gene-related results found in the KEGG and GO pathways. The mutational alterations were also analyzed for different miRs, which showed significant alignment scores with SARS-CoV-2 genomes from other geographical regions. Concomitantly, we also analyzed and compared Bioproject PRJNA615032 trancriptome data obtained from normal vs SARS-CoV-2-infected lung tissue biopsy samples. Data was submitted by tenOever Lab, Microbiology, Icahn School of Medicine at Mount Sinai on 24-Mar-2020. In addition, the gene expression differences between A549 and NHEB cell line data and the gene expression differences between normal and infected lung tissue samples were analyzed in the REACTOME database (https://reactome.org/) to propose the potentially affected signaling axis members. The clinical outcome of severe condition patients is discussed below, for selected miRs and their potential biological importance in host cells.

### 4.1. Biological Significance of Top Ranked miRs in Humans

#### 4.1.1. miR-8066

Recently, the N nucleocapsid gene-related putative miR candidates were shown through *in silico* prediction tools [38]. In a similar manner, we determined miR-8066, a mature sequence found on SARS-CoV-2 genomes, in SARS-CoV-2 genome alignment analysis with human miRs. Additionally, miR-8067, which possesses a similar biological role to miR-8066, was identified as a stem loop region sequence in all SARS-CoV-2 genomes (Figure S1). Previous reports showed that both miRs are found in plasma samples of sepsis patients with severe clinical outcomes [39]. Similar miR family members such as miR-8054, miR-8057, miR-8061, and miR-8068, were also found in a non-survivor group of sepsis patients. It has been shown by in silico analysis that certain miRNAs have the core motifs of AUUGUUG, and that miR-8066 is one of these [40]. When miRs have this motif, it increases their likelihood to bind and activate Nf κB-mediated TLR-8 expression and induce cytokine synthesis [40]. miR-8066 was identified at high levels in tissue biopsies as well as in exosomes [41]. Therefore, SARS-CoV-2-mediated alterations of miRs may act as autocrine or paracrine agonists of host cells to trigger pro-inflammatory cytokines, due to their increased NfKB activity [42]. Similar findings were also suggested for cancer cells, which showed miR alterations in association with inflammation markers [43].

There is preliminary data about the existence of sequence similarity of human miRs in the spike region of the SARS-CoV-2 genome for both forward and reverse complementary strands [44]. Hsa-miR-8055, which was also previously shown to be a sepsis marker, was found to be involved in T-cell responses to specific antigens [45]. One of the differentially expressed circRNAs in children with fulminant myocarditis, Circ_0071542, was suggested to regulate the expression of MAPK, a well-characterized target in the development of disease through binding hsa-miR-8055 [45]. In our current study, we found that the miR-8066 sequence is present in four different SARS-CoV-2 genomes and shows strong association in KEGG pathways for TGF-beta signalling, mucin type O-glycan biosynthesis and cytokine-cytokine receptor interaction. These targeted pathways are known, critical mediators for the clinical outcomes for SARS-CoV-2-infected patients and give us insight about virus pathophysiology. It is well established that for enveloped viruses, such as SARS-CoV-2, N- or O-glycosylation of S protein determines the viral entry and membrane fusion with functional elicitation of host immune responses [46]. The high-resolution LC-MS/MS experiment was performed to detect site-specific quantitative N-linked and O-linked glycan profiling on SARS-CoV-2 subunit S1 and S2 proteins. The glycan profiling showed that two unexpected O-glycosylation sites on the receptor-binding domain (RBD) of subunit S1 increased the pathogenicity of SARS-CoV-2. Therefore, our understanding of complex sialylated N-glycans and sialylated mucin type O-glycans on the functional RBD domain may help to evaluate better therapeutic or vaccine strategies [47]. Moreover, miR-8066 alters N-glycosylation patterns according to the microT-CDS pathway and may have biomarker potential for this mechanism. In addition, miR-8066 is found to be associated with one of the critical clinical problems of COVID-19, the cytokine storm, owing to its potential effect on the cytokine-cytokine receptor pathway. It is well established that miR-8066 affects PRLR, CXCL6, IL6, IL17 and ACVR1 target genes, which are crucial members of the cytokine regulatory network (Table 2). According to previous NGS platform results obtained from healthy versus SARS-CoV-2-infected lung tissue biopsies (Bioproject PRJNA615032), the most highly upregulated pathways are chemokine binding receptors (*p* value 7.94 × 10^−4^), FGFR activation pathways (*p* value 0.002) and its downstream pathway (*p* value 0.001), neutrophil degranulation (*p* value 0.003), and IL10 (*p* value 0.005), when compared with normal lung tissue biopsies (Table S2). On the contrary, according to the REACTOME pathway analysis of Bioproject PRJNA615032 data, neutrophil degradation was also downregulated in SARS-CoV-2-infected biopsy specimens (*p* value 1.74 × 10^−6^). Briefly, the downregulation of host cell responses’ related pathway members such as SRP-dependent co-translational protein targeting to membrane, L13a-mediated translational silencing of ceruloplasmin expression antigen processing, ribosomal organization, and the ERAD pathway, were observed in COVID-19-positive lung biopsy specimens. All of these preliminary gene expression differences, between normal and SARS-CoV-2-infected lung biopsies, are associated with detected miR pathways and highlight common clinical presentations of patients. Each of these biological mechanisms, according to potential disease progression-related alterations in the host genome, is highly associated with TGF-β, which is a cytokine affecting a number of host responses during infection [48]. TGF-β mediates the immune responses of host cells, as well as altering tissue remodelling by affecting cell survival, apoptosis and migration. It has been demonstrated that SARS-CoV N protein potentiate Smad-3 mediated TGF-β activation, and plasminogen activator inhibitor-1 (PAI-1), leading to severe pulmonary fibrosis and inactivation of pro-apoptotic genes by complex formation of Smad3 and Smad4 [48].

#### 4.1.2. miR-5197

miR-5197-3p was identified as the most effective miR to interact with the guide RNA of SARS-CoV, MERS-CoV and COVID-19 [49]. According to a recent study, out of 2565 miRNAs, only three critical miRs, 5197-3p, 4778-3p and 6864-5p could interact with complete complementary miR (cc-miR) and possess a critical therapeutic potential due to their binding affinity on SARS-CoV-2 guide RNA. It was suggested that the generation of miR-5197-3p-based complete complementary miRNA may possess a significant therapeutic response, owing to its structural affinity to guide RNA of SARS-CoV-2, without any side effects on human genes [49]. Previous patents on hsa-miR-5197-5p indicated that any drug targeting this miR might be critical in the treatment of hepatitis B infections (WO2018193902A1). Similar to this finding, it was shown that miR-5197-3p might be used in vaccine strategies in the herpes simplex virus (HSV-1) (WO2013109604A1WO2013109604A1).

In another study, allelic variances of hsa-miR-5197 were found to be highly associated with non-small cell lung cancer (NSCLC) [50]. According to the Harvard and MD Anderson databases, miR-5197 shows significant differences between Caucasians and Chinese populations (data gained from a Han Chinese cohort from Nanjing, China) [50]. The rs2042253 polymorphism (T>C variation) of miR-5197 provided a protective effect on lung cancer survival. Moreover, this SNP is reported to have a high read frequency for paediatric acute lymphoblastic leukemia (ALL) in the UK cohort [51]. High throughput analysis of 408 lung cancer patients showed that miR-5197 polymorphism is highly correlated with chemotherapy-induced severe toxicity [52]. It has been accepted that nucleotide variations in pre-miRNAs may have contributed to the stability of stem-loop structure and may affect recognition sites for Drosha and Dicer cleavage [53]. In our current study, we did not find any mutations in miR-5197 sequences obtained from different SARS-CoV-2 genomes, however we found that **mucin type O-glycan biosynthesis** was related to both miRs 5197-3 and 8066. This KEGG pathway has been involved in the cell-to-cell transmission of human T cell leukemia virus type 1 (HTLV-1) [54] and plays roles in Ebola virus cell attachment [55]. HIV-1 infection of T cells has also been shown to result in altered glycosylation of cell surface glycoproteins [56].

O-glycans are also linked to avian influenza [57]. Mucosal O-linked glycans are furthermore associated with herpes simplex virus 1 infection [58]. Mucin type O-glycan biosynthesis miR pathways have been identified in avian oncogenic retrovirus (Avian leukosis virus subgroup J (ALV-J)) [59]. This KEGG pathway is therefore related both to human as well as veterinary viral infections and further studies will be needed to establish the function of miR-5197 during SARS-CoV-2 infection.

#### 4.1.3. miR-3611

In our current study, we found that miR-3611 is a positive hit for stem loop region and mature miRNA prediction. Because of its potential presence in SARS-CoV-2-mediated cellular responses, we have analysed data from the lung tissue biopsy database (PRJNA615032 Bioproject) to understand its function. A previous study reported altered miRNA expression in chronic obstructive pulmonary disease (COPD). COPD is an airway disorder and respiratory disease that is associated with persistent inflammation [60]. A study on miR expression analysis, conducted on COPD and healthy volunteers, showed significant down-regulation of miR-3611 expression in COPD patients [61]. The long non-coding RNA, H19, has been linked to many carcinomas, including lung cancer [62]. It has been shown that miR-3611 is significantly down-regulated in a H19 knockdown lung cancer cell line (SPC-A1), which indicated overexpression of this miR in ‘normal’ H19 intact lung cancer cell lines [63]. In our study, we identified that **miR-3611** shows high similarity with all four main SARS-CoV-2 genomes and we did not detect any mutations between genomes. However, **miR-3611** was strongly associated with KEGG pathways for metabolism of xenobiotics by cytochrome P450, morphine addiction and GABAergic synapse. Moreover, **morphine addiction** was, besides linkage with **miR-3611**, also linked to **miR-5197-3**. This KEGG pathway has been related to enhanced HIV-1 infection [30] and morphine treatment has been shown to promote HIV-1 replication in macrophages via inhibition of the TLR9 pathway [64]. Furthermore, an increased rate of HIV-1/HTLV-I infection has been observed due to morphine in injection drug users [65]. Morphine is also associated with enhanced hepatitis C virus (HCV) replicon expression [66,67]. Opioids have furthermore been shown to enhance simian acquired immunodeficiency syndrome (SAIDS) in rhesus monkeys [68], while reduced clearance of pulmonary influenza virus infection was observed in morphine-treated Lewis rats [69]. Interestingly, opioids such as morphine are also used as indirect antitussives to suppress cough, which is commonly associated with respiratory viral infections [70], including SARS-CoV-2. Indeed, morphine was used in recent cases in China during sedation and analgesia for endotracheal intubation, to avoid patients’ cough and agitation during the procedure [39]. In light of the association of morphine with promotion of viral replication, it may have effects in SARS-CoV-2 that need to be further investigated.

#### 4.1.4. miR-3934-3p

miRNA expression profiles have been used to classify cancers into various subtypes. miR3934 is found upregulated in colon cancer and was suggested as a biomarker for lung cancer as its expression correlated with survival rate and prognosis of NSCLC [71,72]. Moreover, it has been reported that miR-3934-5p expression significantly increases in NSCLC cell line A549 [73]. It is also a SNP linked to TGF-β signalling and has been identified as downregulated in rectal carcinoma mucosa, compared with normal mucosa [74].

In addition, it was shown that miR-3934-3p downregulated TGFBR1 and SMAD3. In a similar vein, HSV-1 viral infection led to a significant down-regulation of these targets [74]. Moreover, the activation of the TGF-β/Smad pathway is critical for lung fibrosis, which was previously shown in SARS-CoV-related cases. Dysregulation of ACE2 may influence the toll-receptor signalling pathway, via IL6, and affect downstream immune responses. Irrespective of SARS-CoV-2 or pneumonia in TCGA-LUAD, the altered immunoreaction was the primary cause (lung adenocarcinoma; SARS-CoV-2; ACE2; miR-125b-5p; IL6). TGF-β and cigarette smoke have been shown to suppress miR-141-5p to promote CCR5 expression on primary bronchial epithelial cells, which results in increased viral entry and infection by R5-tropic HIV [75]. Given that TGF-β signalling is upregulated by trans-activator (Tat) protein, cigarette smoke and in chronic lung diseases, it has been determined the effects of persistent TGF-β signalling on HIV infection in primary bronchial epithelium re-differentiated *ex vivo* [75]. In our current study, miR-3934-3p was found to be associated with KEGG pathways for glycosaminoglycan biosynthesis heparan sulfate/heparin, mucin type O-glycan biosynthesis and vitamin digestion and absorption. The relevance of these pathways is as follows:

“**Glycosaminoglycan** biosynthesis—heparan sulfate/heparin” was here related to miRs 3934-3. Heparan sulfate proteoglycans have previously been identified to provide the binding sites for SARS-CoV-2 invasion at the early attachment phase [76]. Furthermore, human coronavirus NL63 has been shown to utilise heparan sulfate proteoglycans for target cell attachment [77]. This KEGG pathway has been related to Ebola virus, where heparan sulfate has been identified as an important mediator in polarised epithelial cells [78,79]. In hepatitis C viral infection, the virus hijacks this pathway via interaction with apolipoprotein E for cell entry [63], while heparan sulfate proteoglycans are required for cellular binding of the hepatitis E virus ORF2 capsid protein and for viral infection [70]. Endogenous HERV-K furthermore binds to heparin for cell entry [80], and, while heparin has been found to further Zika virus infection, it acts as an antiviral against Dengue replication [81]. Heparin sulphate is identified as an inhibitory regulator of porcine epidemic diarrhoea virus infection [82] and acts as an attachment factor for rabies virus entry and infection [83], as well as an enhancer of Nipah and Hendra virus infections, which are highly pathogenic, zoonotic paramyxoviruses [84]. This highlights the importance of this KEGG pathway both in human, zoonotic and veterinary viral infections.

Another important pathway, **vitamin digestion and absorption**, was found to be related to miR-3934-3. This KEGG pathway has been highlighted amongst others as a link between dysbiosis of the gut microbiome and chronic diseases [85]. Furthermore, intestinal triglyceride-rich lipoproteins have been related to vitamin metabolism in relation to coronary artery disease as well as viral infections [86]. In AIDS, malabsorption of vitamin 12 has been related to gastric secretory failure, including chronic diarrhoea, due to advanced HIV infection [87,88]. Additionally, vitamin A deficiency has been associated with more progressive HIV disease [89]. Veterinary viral infections, such as infectious bronchitis virus (IBV) and reovirus (RV) in chickens, have been identified to affect vitamin A metabolism due to epithelial damage [90]. Interestingly, a link between vitamin D deficiency and SARS-CoV-2 infection severity was recently reported [91]. Also, vitamin B3 was found to be highly effective to help lung tissue damage repair [92] and it was suggested to be given to COVID-19 patients as soon as the CT lung abnormalities were detected [93].

#### 4.1.5. miR-1307-3p 

miR-1307 has previously been shown to be one of the lung-tissue-associated miRs [94] and was reported as an especially important target in lung development in newborns. Expression of mir-1307-3p has furthermore been linked to acid metabolism in response to aspirin in human cardiac and peri-cardiac fat-derived mesenchymal stem cells (MSCs) [95]. Moreover, it was shown that several miRs, including miR-1307-3p, are involved in TGF-β and semaphorin signalling, as well as inflammatory responses. miRs play an important role, especially during lung morphogenesis in the early stages of development. Interestingly, miR-1307 has been associated with the severity of pulmonary hypertension in systemic scleroderma [96]. Persistent problems with SARS-CoV-2-infected patients, such as oxygen dependency, urgent need for mechanical ventilation, persistent wheezing, and increased risk for pulmonary infections correlate with involvement of miR-1307-3p, as indicated above, due to its role in pulmonary hypertension and chronic lung diseases. The TGF-β superfamily plays critical roles in pre- and postnatal lung development, importantly shaping alveolarisation and controlling the extracellular matrix composition and tissue homeostasis, among other functions. TGF-β signalling is therefore strongly linked to both pulmonary and cardiovascular diseases [97,98,99].

#### 4.1.6. miR-3691-3p 

It has been shown that oxidative stress, which has detrimental effects on intercellular communication, plays an important role in many lung pathologies such as acute lung injury and COPD. A proteomic profile of exosomes from human bronchial epithelial cells, under normoxia and hypoxia, has reported reduced expression of miR-3691-3p in hypoxia [100]. Interestingly, miR3691-3p targets several cell signalling pathways, such as TGF-β signalling, FGF2 and also VCAM1, which is relevant for lung injury and repair. In the current study, we have also identified that miR3691-3p exists in four of the genomes, which we have extensively studied here, and therefore it seems to be a conserved miR.

#### 4.1.7. miR1468-5p

Sudden cardiac death is a major problem amongst the unexplained deaths in COVID-19, and it has been identified that many of those patients suffered from primary myocardial fibrosis (PMF), without any known aetiology. Recently, higher expression of miR-1468-3p was identified as a disease-associated and age-dependent cardiac biomarker, as it promotes cardiac fibrosis and cell senescence, although no difference was noted in the mature form of miR-1468 between healthy and COVID-19-diseased cardiac tissue [101]. TGF-β1 plays a key role in fibrosis-related pathologies including cardiac fibrosis, and, furthermore, miR-1468 activates non-canonical TGF-β1 and MAPKs signalling pathways [101]. Moreover, miR-1468-5p expression has been found to be upregulated in regulatory T cells, which have a significant role in autoimmune disorders, transplant rejection, allergic diseases, and asthma [102]. miR-1468-5p has previously been associated with glioma, where it inhibits growth and cell cycle progression by targeting ribonucleotide reductase large subunit M1 (RRM1), based on a study on patients from the Chinese Glioma Genome Atlas [103]. miR-1468-5 is also linked to progressing hepatocellular carcinoma [104]. Interestingly, in Alzheimer’s disease, miR-1468-5p has been identified to be at lower abundance compared with healthy controls [105]. It has furthermore been identified as a biomarker in late seizure in patients with spontaneous intracerebral haemorrhage [49]. The link between miR-1468-5 in viral infection and other comorbidities will need to be further investigated.

**miR-129-2-3p**, here identified as a common mutated miR, has previously been identified as a regulator in human cancer development and progression [106]. It has been identified as a diagnostic and prognostic biomarker for renal cell carcinoma [107] and a suppressor of serous ovarian cancer [104]. Its upregulation suppresses breast cancer cell proliferation and induces its apoptosis, while downregulation, via hypermethylation, increases breast cancer progression due to BCL2L2 overexpression [107]. It furthermore attenuates cell migration and invasion in renal cell carcinoma by affecting the downregulation of various metastasis-related genes [108]. miR129-2 has been linked to a range of haematological malignancies, including lymphoma [109]. It is also linked to lung adenocarcinoma including regulation of cell proliferation [110], as well as to hepatocellular carcinoma [111]. Interestingly, miR-129-2-3p has been found to be upregulated in human papilloma virus-positive (HPV) head and neck squamous cell carcinoma [112] and in HPV transfected keratinocyte cells [113]. In the light of increasing understanding of the link between SARS-CoV-2 and comorbidities, underlying changes in miR-129-2-3p expression may be of considerable importance.

**A number of KEGG pathways** have been strongly linked to the main miRs identified as being related to sequences within the SARS-CoV-2 genomes. These include: “Mucin type O-glycan biosynthesis”, “TGF-β signalling pathway”, “Morphine addiction”, “Metabolism of xenobiotics by cytochrome P450”, “Other types of O-glycan biosynthesis”, “Vitamin digestion and absorption”, “Glycosaminoglycan biosynthesis—heparan sulfate/heparin”, “GABAergic synapse”, “Cytokine-cytokine receptor interaction”, “Signalling pathways regulating pluripotency of stem cells”, “Amphetamine addiction”, “Axon guidance”, “Hippo signalling pathway”, “Prolactin signalling pathway”, “mRNA surveillance pathway”, “Glycosphingolipid biosynthesis—lacto and neolacto series”, “Bile secretion”, “Circadian entrainment”, “N-glycan biosynthesis”, “Mismatch repair”, “Drug metabolism—cytochrome P450”, “Glutamatergic synapse”, “Glycosaminoglycan degradation”, “Antigen processing and presentation”. The relevance of several of these KEGG pathways has been discussed above in direct relation to the various miRs and provides a novel insight into the putative interplay of these pathways and the microRNAs identified in COVID-19, and may also help in furthering understanding of the interplay of miRs, viral infections and comorbidities.

## 5. Conclusions

Previous epidemics have been caused by *betacoronaviruses*, especially in Asia. As expected, several similarities and differences in the epidemiology, clinical features, and management of SARS, MERS, and SARS-CoV-19 were seen [114]. However, none of the previous infections has caused global pandemics of the scale currently caused by SARS-CoV-2. In our study, analysis of several SARS-CoV-19 genomes, which were isolated from different geographical regions, shows significant similarity scores with human miRs, which target a subset of genes related to pathways affecting virus pathogenicity and host responses observed in COVID-19 patients. The numbers of *in silico* prediction-based studies that show miRNA mimicking sequences of SARS-CoV-2 genome and miR-mediated host responses are increasing. It was suggested that, as hsa-miR-4661-3p may target the S gene of SARS-CoV-2, and a virus-encoded miRNA miR147-3p could enhance the expression of TMPRSS2 to promote SARS-CoV-2 infection in the gut, host miRs are critical in the progression of the disease. In our current study we identified that seven completely complementary miRNAs of COVID-19 (cc-miRc) prevent viral replication and protein translation processes. Similar predictions and biological proofs were determined for MERS and SARS-CoV [115,116].

By way of comparison to other human coronaviruses, multiple sequence alignment was carried out, comparing the four SARS-CoV-2 sequences with those from SARS, MERS and two cold viruses, OC43 and 229E (Table S3), [115]. The symptom spectrum for these viruses differ from that of SARS-CoV-2, therefore it is instructive to see if the miR-like sequences are present. We identified seven key miRs, which highlight considerable differences between the SARS-CoV-2 sequences, compared with the other viruses (Table S2). In each case, the four SARS-CoV-2 sequences are identical, but, for the most part, the level of conservation in the other genomes is poor. Across the four sequences, which for SARS generally show the highest degree of conservation: one ‘miR’ is completely conserved (miR-1468), while others range from two to ten nucleotide differences. Whilst it is possible that this decrease in similarity could result in reduced levels of transcriptional control, it is clear that there will be a change in the physiological effect of the virus. The proposed miR1307 has been suggested as a therapeutic target in the prevention of SARS-CoV-2 infection. Therefore, more studies on *in silico* patterns within the SARS-CoV-2 genome may provide a deeper understanding about miR-based novel therapeutics [117,118]. MERS shows greater divergence across these segments and the two milder symptom viruses show even greater differences or even significant sequence gaps. This divergence away from the SARS-CoV-2 sequences broadly mirrors the phylogenetic relationships obtained from the whole-genome alignments. Thus it can be argued that patterns of mutation, occurring during sequence divergence from the longer established human viruses to the more recent ones, have led to the emergence of sequence motifs that can be related directly to the pathogenicity of SARS-CoV-2.

This mechanism could have considerable significance in determining the symptom spectrum of future potential pandemics. KEGG pathway analysis revealed a number of critical pathways linked to the seven identified miRs that may provide insight into the interplay between the virus and comorbidities.

Based on our reported findings, miRs may constitute a potential and effective therapeutic approach to cure COVID19 infection and its pathological consequences, requiring further validation in large cohort patient-derived samples.

## Figures and Tables

**Table 1:** Similar microRNA (miR) sequences found in SARS-CoV-2-released genomes from different geographical areas.

miRs	Score	E-Value	Alignment	Wuhan	Italy	UK	Valencia	Turkey	Vero E6
				NC_045512.2	MT066156.1	hCoV-19/England/20136087804/2020|EPI_ISL_420910	MT198652.2	hCoV-19/Turkey/GLAB-CoV008/2020	hCoV-19/Turkey/ERAGEM-001/2020
hsa-miR-8066	80	1.6–2.8	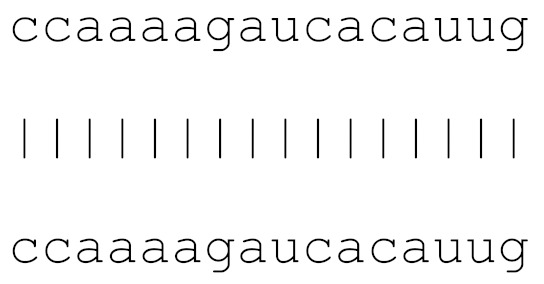	√	√	√	√	√	√
hsa-miR-5197-3p	79	1.6–2.8	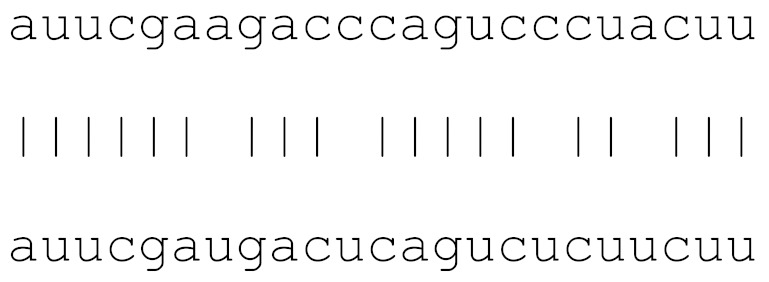	√	√	√	√	√	√
hsa-miR-3611	77	2.8–3.8	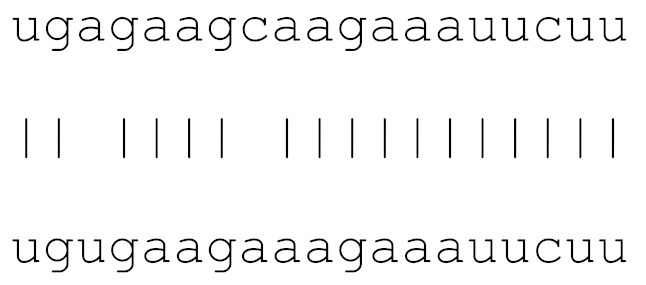	√	√	√	√	√	√
hsa-miR-3934-3p	76	3.4–5.0	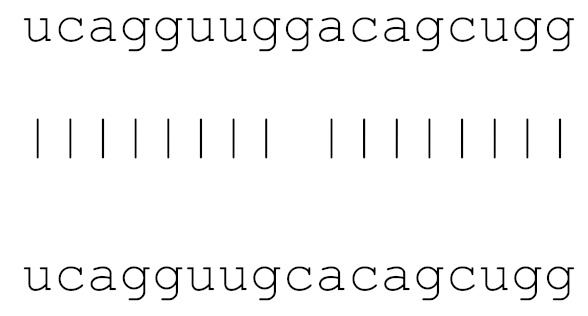	√	√	√	√	√	√
hsa-miR-1468-5p	71	4.7–8.8	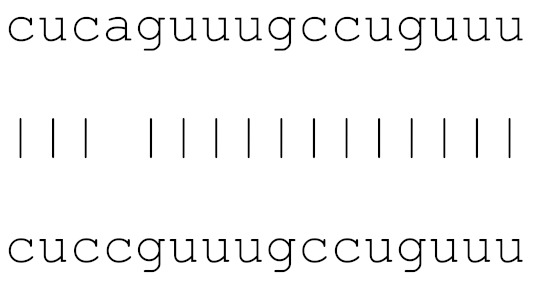	√	√	√	√	√	√
hsa-miR-1307-3p	72	4.3–6.3	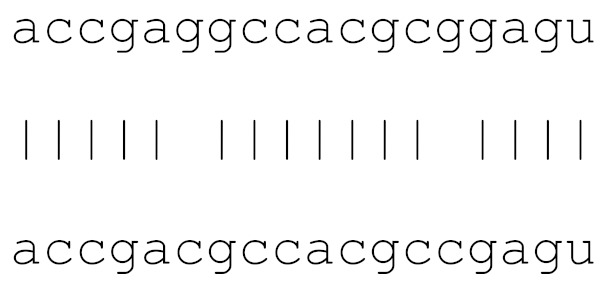	√	√	√	√	√	
hsa-miR-3691-3p	74	5.0–9.5	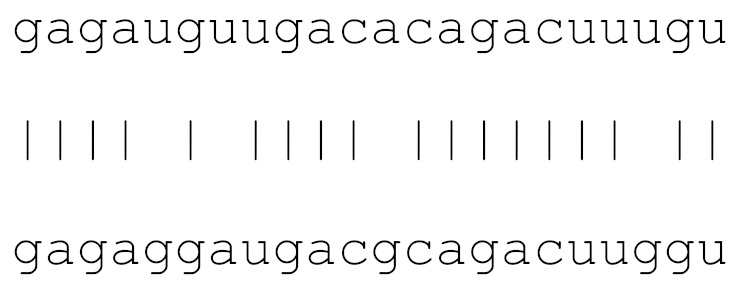	√	√	√	√	√	
hsa-miR-3120-5p	73	6.0–7.2	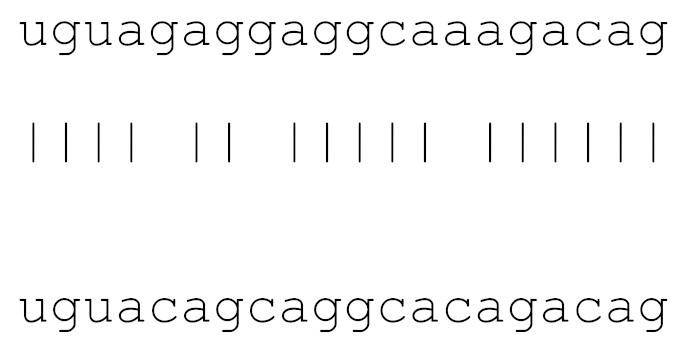		√	√	√	√	√
hsa-miR-3914	73	6.0–8.5	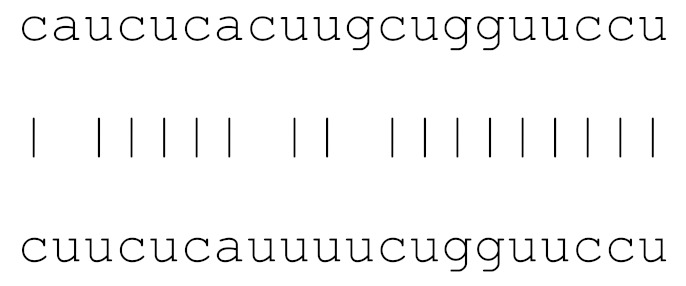		√	√	√	√	√
hsa-miR-3672	72	7.3–9.8	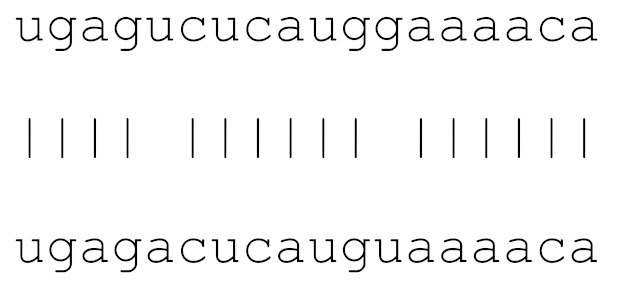		X	X	X		X
hsa-miR-7107-3p	73	6.0–6.2	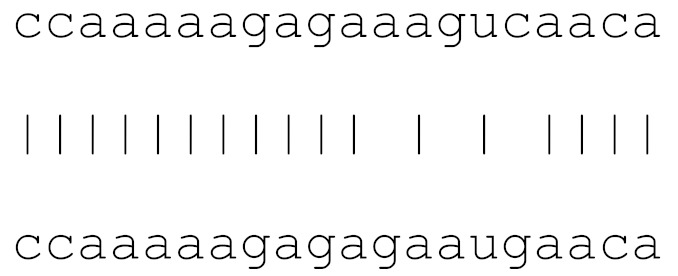	√	√	√		√	√
hsa-miR-1287-5p	73	6.0–8.3	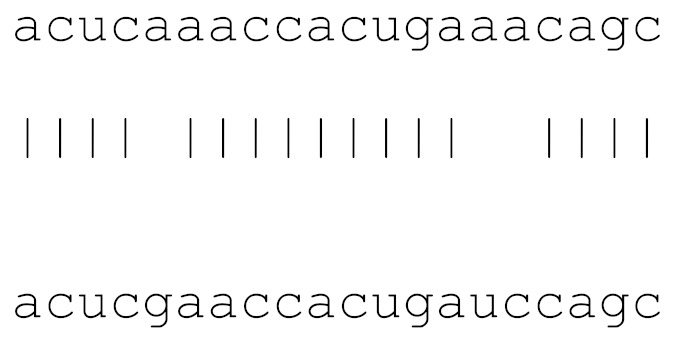	√	√	√		√	√
hsa-miR-129-2-3p	73	6.0–7.7	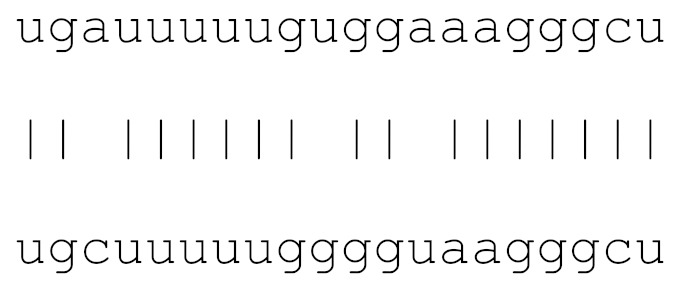	√	√				√
hsa-miR-378c	71	8.8–9.3	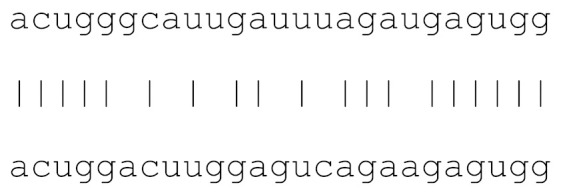		√		√		√
hsa-miR-10397-5p	72	6.9–10.0	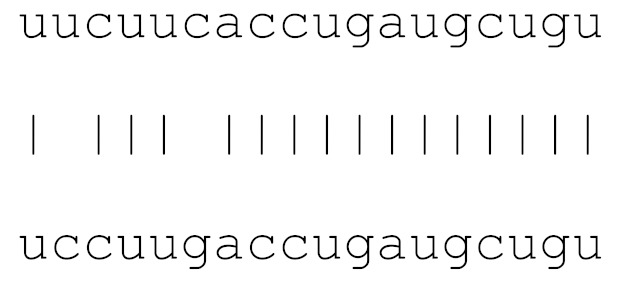		√	√			√
hsa-miR-584-3p	72	7.3–9.8	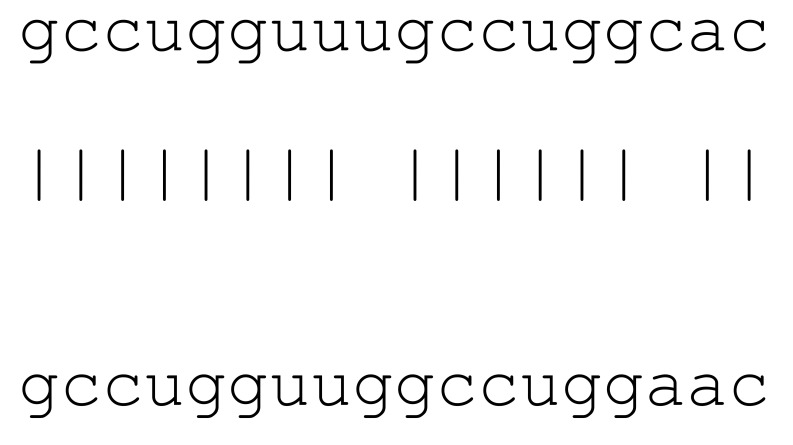		√	√			
hsa-miR-3085-3p	71	8.8–9.9	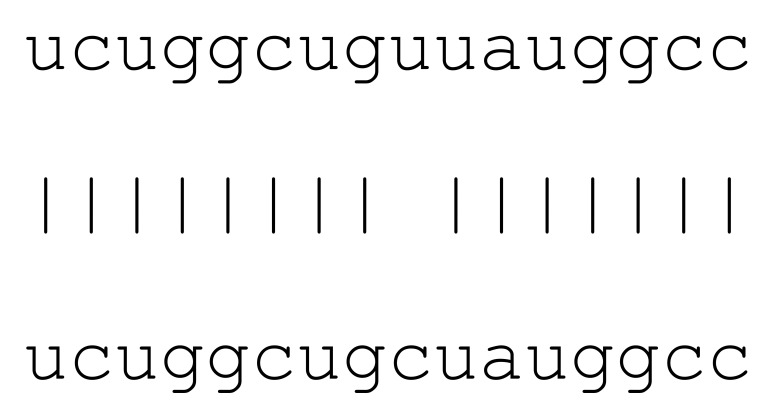		√	√			√
hsa-miR-3191-3p	70	7.4–8.5	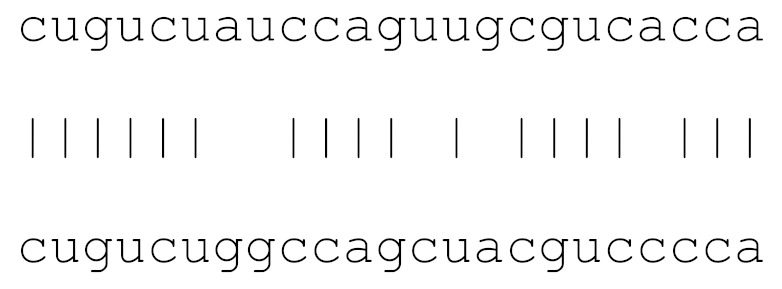				√		√
hsa-miR-148b-3p	72	8.2–9.8	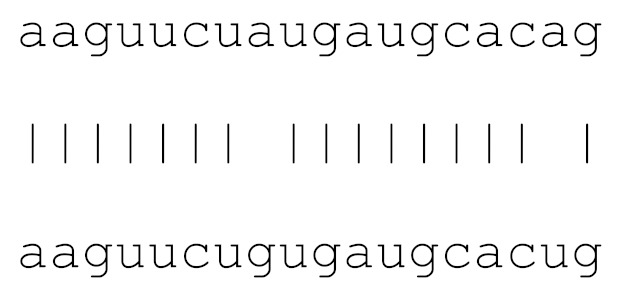				√		√
hsa-miR-3529-3p	69	9.0	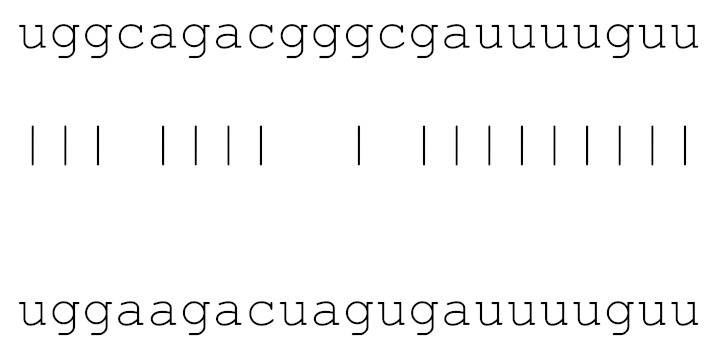				√		
hsa-miR-3682-5p	68	9.0	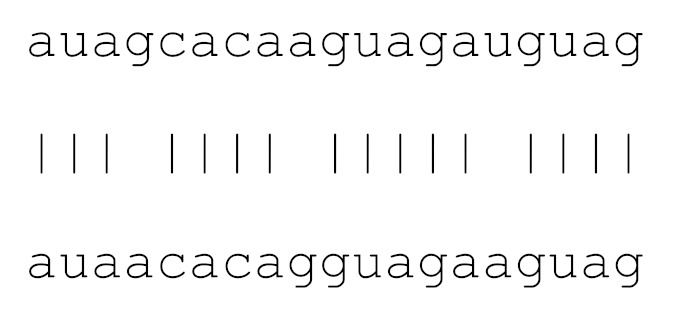				√	√	

**Table 2:** The KEGG (A) and GO (B) enrichment analysis results for miR-8066, 5197-3p, 3611, 3934-3p, 1468-5p, 3691, and 1307-3p.

**KEGG Pathway (A)**	***p*-Value**	**#genes**	**#miRNAs**
Mucin type O-Glycan biosynthesis	2.52 × 10^−2^	7	3
TGF-beta signaling pathway	4.96 × 10^−1^	12	4
Morphine addiction	0.0001128919	14	5
Metabolism of xenobiotics by cytochrome P450	0.0002215491	5	2
Other types of O-glycan biosynthesis	0.0003646344	1	1
Vitamin digestion and absorption	0.001008222	2	1
Glycosaminoglycan biosynthesis—heparan sulfate/heparin	0.00385809	1	1
GABAergic synapse	0.01342039	13	4
Cytokine-cytokine receptor interaction	0.02096334	9	1
Signaling pathways regulating pluripotency of stem cells	0.180299	9	1
Amphetamine addiction	0.2150865	7	1
Axon guidance	0.2239648	22	3
Hippo signaling pathway	0.2278356	7	1
Prolactin signaling pathway	0.2284669	5	1
mRNA surveillance pathway	0.2795597	1	1
Glycosphingolipid biosynthesis—lacto and neolacto series	0.3157068	1	1
Bile secretion	0.4120997	1	1
Circadian entrainment	0.4608082	9	1
N-Glycan biosynthesis	0.488078	2	1
Mismatch repair	0.6174557	1	1
Drug metabolism—cytochrome P450	0.7063987	6	1
Glutamatergic synapse	0.7319762	6	1
Glycosaminoglycan degradation	0.7395672	2	1
Antigen processing and presentation	0.7591685	1	1
**GO Category (B)**	***p*-Value**	**#genes**	**#miRNAs**
organelle	1 × 10^−38^	848	6
cellular nitrogen compound metabolic process	1 × 10^−12^	414	7
ion binding	8 × 10^−8^	495	7
biosynthetic process	3 × 10^−7^	351	7
nucleic acid binding transcription factor activity	4 × 10^−2^	115	6
cellular protein modification process	2 × 10^1^	205	7
molecular_function	5 × 10^3^	1303	7
cellular_component	1 × 10^5^	1312	7
enzyme binding	2 × 10^5^	119	5
gene expression	3 × 10^5^	54	6
protein binding transcription factor activity	1 × 10^6^	52	5
blood coagulation	0.000176061599974	44	6
protein complex	0.00115693944276	290	5
post-translational protein modification	0.00185490003064	19	5
neurotrophin TRK receptor signaling pathway	0.00197464302174	24	5
synaptic transmission	0.00204631087649	42	5
cellular protein metabolic process	0.00275618650845	39	5
small molecule metabolic process	0.00275618650845	170	7
cytoskeletal protein binding	0.00396272124679	68	4
cell-cell signaling	0.00396272124679	60	5
transcription, DNA-templated	0.00450420995446	208	6
symbiosis, encompassing mutualism through parasitism	0.0140041886634	41	5
catabolic process	0.0141620388146	142	6
Fc-epsilon receptor signaling pathway	0.0222360628043	15	6
cellular component assembly	0.02375306792	99	5
transcription initiation from RNA polymerase II promoter	0.0250016205995	24	5
nucleoplasm	0.0335128910566	92	6
platelet activation	0.0350801245107	20	5
positive regulation of telomere maintenance via telomerase	0.0370638891992	3	3
RNA polymerase II core promoter proximal region sequence-specific DNA binding transcription factor activity involved in positive regulation of transcription	0.0448871926331	32	5
O-glycan processing	0.0449415771561	8	5

**Table 3:** The mutational comparison of selected miRs found on the Wuhan genome compared to other SARS-CoV-2 strains isolated from different geographical regions. % represents the number of viral genome sequences with a single base change in that miR sequence. *n* = number of viral genomes analysed.

miRs	Alignment	Wuhan/China	Italy	Spain	France	England	USA	India
		*n* = 28	*n* = 44	*n* = 133	*n* = 104	*n* = 104	*n* = 104	*n* = 34
**hsa-miR-8066**	ccaaaagaucacauug	0	0	0	0	0	0	0
**hsa-miR-5197-3p**	auucgaagacccagucccuacuu	0	0	0	0	0	0.9%	0
**hsa-miR-3611**	ugagaagcaagaaauucuu	0	0	0	0	0	0	0
**hsa-miR-3934-3p**	ucagguuggacagcugg	0	0	0	0	0	0	0
**hsa-miR-1307-3p**	accgaggccacgcggagu	3.5%	2.2%	8.27%	1.92%	2.88%	2.88%	38.23%
**hsa-miR-3691-3p**	gagauguugacacagacuuugu	0	0	0	0	0	0	0
**hsa-miR-1468-5p**	cucaguuugccuguuu	0	0	2.25%	0.96%	0	0	8.83%
**hsa-miR-3120-5p**	uguagaggaggcaaagacag	0	0	0	0	0	0	0
**hsa-miR-3914**	caucucacuugcugguuccu	0	0	0	0	0	0	0
**hsa-miR-3672**	ugagucucauggaaaaca	0	0	0.75%	0.96%	0	0	0
**hsa-miR-378c**	acugggcauugauuuagaugagugg	0	0	0	0	0	0	0
**hsa-miR-7107-3p**	ccaaaaagagaaagucaaca	0	0	0	0	0	0	0
**hsa-miR-1287-5p**	acucaaaccacugaaacagc	0	0	0	0	0	0	0
**hsa-miR-10397-5p**	uucuucaccugaugcugu	0	0	0	0	0	0	0
**hsa-miR-584-3p**	gccugguuugccuggcac	0	0	0.75%	0	0	0	0
**hsa-miR-3085-3p**	ucuggcuguuauggcc	0	0	0	0	0	0.96%	0
**hsa-miR-3191-3p**	cugucuauccaguugcgucacca	0	0	0	0	0	0	0
**hsa-miR-3529-3p**	uggcagacgggcgauuuuguu	0	0	0	0	0	0	2.94%
**hsa-miR-3682-5p**	auagcacaaguagauguag	0	0	0	0	0	0.96%	0
**hsa-miR-148b-3p**	aaguucuaugaugcacag	0	0	0	0	0	0	0
**hsa-miR-129-2-3p**	ugauuuuuguggaaagggcu	0	0	0	0	0	0	0

**Table 4:** BioProject data analysis for differential gene expression between non-treated and SARS-CoV-2-treated NEHB and A549 cells.

**WikiPathways**	**p-Adj**
Photodynamic therapy-induced NF-kB survival signaling	0
IL-18 signaling pathway	8.6 × 10^−9^
miRNAs involvement in the immune response in sepsis	2.4 × 10^−8^
Cytokines and Inflammatory Response	9.9 × 10^−7^
Lung fibrosis	2.5 × 10^−6^
**BioPlanet**	**p-Adj**
Oncostatin M	0
Interleukin-1 regulation of extracellular matrix	0
Interleukin-5 regulation of apoptosis	0
TNF-alpha effects on cytokine activity, cell motility, and apoptosis	0
Immune system signaling by interferons, interleukins, prolactin, and growth hormones	0
**KEGG**	**p-Adj**
IL-17 signaling pathway	1.3 × 10^−9^
TNF signaling pathway	1.6 × 10^−9^
Legionellosis	3.5 × 10^−9^
Rheumatoid arthritis	5.4 × 10^−9^
Cytokine-cytokine receptor interaction	6.6 × 10^−9^
**PANTHER**	**p-Adj**
Plasminogen activating cascade	0.00156
Toll receptor signaling pathway	0.00911
CCKR signaling map ST	0.02550
Apoptosis signaling pathway	0.10282
Blood coagulation	0.10433
**REACTOME**	**p-Adj**
Interferon alpha/beta signaling	1.6 × 10^−9^
Interleukin-10 signaling	2.5 × 10^−9^
Interleukin-4 and Interleukin-13 signaling	2.4 × 10^−7^
Formation of the cornified envelope	1.3 × 10^−5^
Chemokine receptors bind chemokines	0.00047
**Small Molecule Pathway DB**	**p-Adj**
CD40L Signalling Pathway	0.25268
NF-kB Signaling Pathway	0.25268
Toll-Like Receptor Pathway 2	0.25268
Capecitabine Metabolism Pathway	0.25268
Capecitabine Action Pathway	0.25268
**BIOCYC**	**p-Adj**
vitamin D3 biosynthesis	0.03597
guanosine nucleotides degradation	0.03597
retinoate biosynthesis II	0.03597
guanosine nucleotides degradation III	0.03597
adenosine nucleotides degradation II	0.03597
**Pathway Interaction DB**	**p-Adj**
Validated transcriptional targets of AP1 family members Fra1 and Fra2	3.8 × 10^−5^
IL23-mediated signaling events	0.00050
CD40/CD40L signaling	0.02539
Glucocorticoid receptor regulatory network	0.02603
LPA receptor mediated events	0.04171

## Supplementary Materials

The following are available online at https://www.mdpi.com/1999-4915/12/6/614/s1. Table S1. List of the SARS-CoV-2 strains isolated from different geographical regions that included for the conservation of miR mimic sequences. Table S2.Upregulated pathways from SARS-CoV-2 infected lung tissue biopsies when compared to the healthy lung biopsies, obtained from Bioproject PRJNA615032. Table S3. Segments spanning the putative viral miR sequences were taken from a complete genome multiple sequence alignment of geographically different SARS-CoV-2 and other coronaviruses. Figure S1. miR8067 structure is drawn with miREval algorithm (http://mimirna.centenary.org.au/mireval/) as a stem loop prediction sequence found on the SARS-CoV-2 genome.
