# Supplementary material for: The Prediction of miRNAs in SARS-CoV-2 Genomes: hsa-miR Databases Identify 7 Key miRs Linked to Host Responses and Virus Pathogenicity-Related KEGG Pathways Significant for Comorbidities
Source: Viruses. 2020 Jun 4;12(6):614. doi: 10.3390/v12060614 (PMC7354481; doi:10.3390/v12060614)
Supplement: Supplementary file 1 [file viruses-12-00614-s001.zip › viruses-826604.suppl zip/Suppl Table 3_Arisan et al 2020.docx]

**Supplementary Table 3.** Segments spanning the putative viral miR sequences taken from a complete genome multiple sequence alignment of geographically different SARS-CoV-2 and other coronaviruses. The alignment was carried out using Clustal Omega at EBI.

**miR-5197**

NC_002645.1 ATTTCGGTGAGAACGAAGAAATTATCATCAATGGTCATACTTACGTTTGTGCTTGGCTTA 767 ***229E***

NC_006213.1 ------------------------------------------------------------ 1062 ***OC43***

KT225476.2 GTGCTGATGACGAAGGCTTCATCACATTAAAGAACAATCTATATAGATTGGTTTGGCATG 1035 ***MERS***

NC_004718.3 TCGAGTCGAAGAGAGGTGTCTACTGCTGCCGTGACCATGAGCATGAAATTGCCTGGTTCA 988 ***SARS***

420910 ttgacactaagaggggtgtatactgctgccgtgaacatgagcatgaaattgcttggtaca 989 ***Covid 19*** ***England***

MT198652.2 TTGACACTAAGAGGGGTGTATACTGCTGCCGTGAACATGAGCATGAAATTGCTTGGTACA 935 ***Covid 19*** ***Valencia***

NC_045512.2 TTGACACTAAGAGGGGTGTATACTGCTGCCGTGAACATGAGCATGAAATTGCTTGGTACA 989 ***Covid 19*** ***Wuhan***

MT066156.1 TTGACACTAAGAGGGGTGTATACTGCTGCCGTGAACATGAGCATGAAATTGCTTGGTACA 989 ***Covid 19*** ***Italy***

NC_002645.1 CTAAGCGT---------------------------------------------------- 775

NC_006213.1 -------------------CTTGGAGTCAGGAACTCCTTTTTGATGTAATTGTGGCATGG 1103

KT225476.2 TTGAGCGTAAAGACGTTCCATATCCTAAGCAATCTATTTTTACTATTAATAGTGTGGTCC 1095

NC_004718.3 CTGAGCGCTCTGATAAGAGCTACGAGCACCAGACACCCTTCGAAATTAAGAGTGCCAAGA 1048

420910 cggaacgttctgaaaagagctatgaattgcagacaccttttgaaattaaattggcaaaga 1049

MT198652.2 CGGAACGTTCTGAAAAGAGCTATGAATTGCAGACACCTTTTGAAATTAAATTGGCAAAGA 995

NC_045512.2 CGGAACGTTCTGAAAAGAGCTATGAATTGCAGACACCTTTTGAAATTAAATTGGCAAAGA 1049

MT066156.1 CGGAACGTTCTGAAAAGAGCTATGAATTGCAGACACCTTTTGAAATTAAATTGGCAAAGA 1049

**miR-8066**

NC_002645.1 TGGTACCAAGTCTTGGTCTGTTGGTGATTGGACCGGTTTTAAATCCTCTTGTTGCAACGT 1095

NC_006213.1 ----------------TATGCAGATGACTCTATTATTTTACGTCAATATAATTTAGTTGA 1279

KT225476.2 ----------------TATGGTAAGGAGTCACTTGAGAACCCAACCTACATTTACCACTC 1270

NC_004718.3 ----------------TACCCTGTTGCATCTCCACAGGAGTGTAACAATATGCACTTGTC 1217

420910 ----------------tatccagttgcgtcaccaaatgaatgcaaccaaatgtgcctttc 1218

MT198652.2 ----------------TATCCAGTTGCGTCACCAAATGAATGCAACCAAATGTGCCTTTC 1164

NC_045512.2 ----------------TATCCAGTTGCGTCACCAAATGAATGCAACCAAATGTGCCTTTC 1218

MT066156.1 ----------------TATCCAGTTGCGTCACCAAATGAATGCAACCAAATGTGCCTTTC 1218

* * *

**miR-3611**

NC_002645.1 CTAAGCGT---------------------------------------------------- 775

NC_006213.1 -------------------CTTGGAGTCAGGAACTCCTTTTTGATGTAATTGTGGCATGG 1103

KT225476.2 TTGAGCGTAAAGACGTTCCATATCCTAAGCAATCTATTTTTACTATTAATAGTGTGGTCC 1095

NC_004718.3 CTGAGCGCTCTGATAAGAGCTACGAGCACCAGACACCCTTCGAAATTAAGAGTGCCAAGA 1048

420910 cggaacgttctgaaaagagctatgaattgcagacaccttttgaaattaaattggcaaaga 1049

MT198652.2 CGGAACGTTCTGAAAAGAGCTATGAATTGCAGACACCTTTTGAAATTAAATTGGCAAAGA 995

NC_045512.2 CGGAACGTTCTGAAAAGAGCTATGAATTGCAGACACCTTTTGAAATTAAATTGGCAAAGA 1049

MT066156.1 CGGAACGTTCTGAAAAGAGCTATGAATTGCAGACACCTTTTGAAATTAAATTGGCAAAGA 1049

**miR-3934**

NC_002645.1 ------------------------------------------------------------ 223

NC_006213.1 CCATATGAT----------TTGGAGGTGCTACTTCAAGATGCTTTGCAGTCCCGTGAAGC 514

KT225476.2 GCACATCATGTCTTTCGTGGCTGGTGTGATCGCGCAAGGTGCGCGCGGTACGTATCGAGC 325

NC_004718.3 GCATACCTAGGTTTC----GTCCGGGTGTGACCGAAAGGTAAGATGGAGAGCCTTGTTCT 281

420910 gcacatctaggtttt----gtccgggtgtgaccgaaaggtaagatggagagccttgtccc 282

MT198652.2 GCACATCTAGGTTTC----GTCCGGGTGTGACCGAAAGGTAAGATGGAGAGCCTTGTCCC 228

NC_045512.2 GCACATCTAGGTTTC----GTCCGGGTGTGACCGAAAGGTAAGATGGAGAGCCTTGTCCC 282

MT066156.1 GCACATCTAGGTTTC----GTCCGGGTGTGACCGAAAGGTAAGATGGAGAGCCTTGTCCC 282

**miR-1037**

NC_002645.1 GTT-----CCTAATGGCCTGCAACCGTGTGACACTTGCCGTAGCAAGTGATTCTGAAATT 340

NC_006213.1 GTTAATAAGCATGTGGCCTATCAGTTATATATGATTGATCCTGCAGGTGTCTGTCTTGGT 698

KT225476.2 TCT-----CTATGTGCCCATCCGGCTGGCTGGACACACTAGACACCTCCCAGGTCCTCGT 533

NC_004718.3 GCC-----CTATGTGTTCATTAAACGTTCTGATGCCTTAAGCACCAATCACGGCCACAAG 516

420910 gcc-----ctatgtgttcatcaaacgttcggatgctcgaactgcacctcatggtcatgtt 517

MT198652.2 GCC-----CTATGTGTTCATCAAACGTTCGGATGCTCGAACTGCACCTCATGGTCATGTT 463

NC_045512.2 GCC-----CTATGTGTTCATCAAACGTTCGGATGCTCGAACTGCACCTCATGGTCATGTT 517

MT066156.1 GCC-----CTATGTGTTCATCAAACGTTCGGATGCTCGAACTGCACCTCATGGTCATGTT 517

** *

**miR-3691**

NC_002645.1 TGACCGTCCTTTTATGCTT---CATGGGTGGTTGGTTTTTTCCAATTCAAATTACCTTTT 585

NC_006213.1 TGATTTTAAAGTTGAAGATGCTTATGACCAGGTGCATGATGAGCCTAAGGGTAAGTTTTC 910

KT225476.2 TGAGCGAGACAACACCTCTTGCCCTGAGTGGATGGACGATTTTGAGGCGGATCCTAAAGG 811

NC_004718.3 TGACTTAGGTGACGAGCTTGGCACTGATCCCATTGAAGATTATGAACAAAACTGGAACAC 752

420910 tgacttaggcgacgagcttggcactgatccttatgaagattttcaagaaaactggaacac 753

MT198652.2 TGACTTAGGCGACGAGCTTGGCACTGATCCTTATGAAGATTTTCAAGAAAACTGGAACAC 699

NC_045512.2 TGACTTAGGCGACGAGCTTGGCACTGATCCTTATGAAGATTTTCAAGAAAACTGGAACAC 753

MT066156.1 TGACTTAGGCGACGAGCTTGGCACTGATCCTTATGAAGATTTTCAAGAAAACTGGAACAC 753

*** * ** *

NC_002645.1 GGAGGAAT------------TTGATGTTGTCTTCGGTAAGAGAGGTGGTGGTAATGTGAC 633

NC_006213.1 TAAGAAGG------------CTTATGCTTTAATTAGAGGGTATCGTGGTGTTAAACCACT 958

KT225476.2 CAAATATG------------CCCAGAATCTGCTTAAGAAGTTGATTGGCGGTGATGTCAC 859

NC_004718.3 TAAGCATGGCAGTGGTGCACTCCGTGAACTCACTCGTGAGCTCAATGGAGGTGCAGTCAC 812

420910 taaacatagcagtggtgttacccgtgaactcatgcgtgagcttaacggaggggcatacac 813

MT198652.2 TAAACATAGCAGTGGTGTTACCCGTGAACTCATGCGTGAGCTTAACGGAGGGGCATACAC 759

NC_045512.2 TAAACATAGCAGTGGTGTTACCCGTGAACTCATGCGTGAGCTTAACGGAGGGGCATACAC 813

MT066156.1 TAAACATAGCAGTGGTGTTACCCGTGAACTCATGCGTGAGCTTAACGGAGGGGCATACAC 813

* * * * ** *

**miR-1468**

NC_002645.1 ------------------------------------------------------------ 223

NC_006213.1 CCATATGAT----------TTGGAGGTGCTACTTCAAGATGCTTTGCAGTCCCGTGAAGC 514

KT225476.2 GCACATCATGTCTTTCGTGGCTGGTGTGATCGCGCAAGGTGCGCGCGGTACGTATCGAGC 325

NC_004718.3 GCATACCTAGGTTTC----GTCCGGGTGTGACCGAAAGGTAAGATGGAGAGCCTTGTTCT 281

420910 gcacatctaggtttt----gtccgggtgtgaccgaaaggtaagatggagagccttgtccc 282

MT198652.2 GCACATCTAGGTTTC----GTCCGGGTGTGACCGAAAGGTAAGATGGAGAGCCTTGTCCC 228

NC_045512.2 GCACATCTAGGTTTC----GTCCGGGTGTGACCGAAAGGTAAGATGGAGAGCCTTGTCCC 282

MT066156.1 GCACATCTAGGTTTC----GTCCGGGTGTGACCGAAAGGTAAGATGGAGAGCCTTGTCCC 282
