# Supplementary material for: The Prediction of miRNAs in SARS-CoV-2 Genomes: hsa-miR Databases Identify 7 Key miRs Linked to Host Responses and Virus Pathogenicity-Related KEGG Pathways Significant for Comorbidities
Source: Viruses. 2020 Jun 4;12(6):614. doi: 10.3390/v12060614 (PMC7354481; doi:10.3390/v12060614)
Supplement: Supplementary file 1 [file viruses-12-00614-s001.zip › viruses-826604.suppl zip/Supplementary Table 1_Arisan et al 2020.docx]

**Supplementary Table 1.** List of the SARS-CoV-2 strains isolated from different geographical regions that included for the conservation of miR mimic sequences.

| **China / Wuhan** |
| --- |
|  |
| hCoV-19/Wuhan/HBCDC-HB-05/2020\|EPI_ISL_412981\|2020-01-18 |
| hCoV-19/Wuhan/IPBCAMS-WH-02/2019\|EPI_ISL_403931\|2019-12-30 |
| hCoV-19/Wuhan/HBCDC-HB-06/2020\|EPI_ISL_412982\|2020-02-07 |
| hCoV-19/Wuhan/HBCDC-HB-04/2020\|EPI_ISL_412980\|2020-01-18 |
| hCoV-19/Wuhan/WH04/2020\|EPI_ISL_406801\|2020-01-05 |
| hCoV-19/Wuhan/IVDC-HB-envF13-21/2020\|EPI_ISL_408515\|2020-01-01 |
| hCoV-19/Wuhan/IPBCAMS-WH-01/2019\|EPI_ISL_402123\|2019-12-24 |
| hCoV-19/Wuhan/HBCDC-HB-02/2020\|EPI_ISL_412978\|2020-01-17 |
| hCoV-19/Wuhan/HBCDC-HB-03/2020\|EPI_ISL_412979\|2020-01-18 |
| hCoV-19/Wuhan/WIV05/2019\|EPI_ISL_402128\|2019-12-30 |
| hCoV-19/Wuhan/WIV07/2019\|EPI_ISL_402130\|2019-12-30 |
| hCoV-19/Wuhan/WH01/2019\|EPI_ISL_406798\|2019-12-26 |
| hCoV-19/Wuhan/IVDC-HB-05/2019\|EPI_ISL_402121\|2019-12-30 |
| hCoV-19/Wuhan/IVDC-HB-04/2020\|EPI_ISL_402120\|2020-01-01 |
| hCoV-19/Wuhan/WIV02/2019\|EPI_ISL_402127\|2019-12-30 |
| hCoV-19/Wuhan/HBCDC-HB-02/2019\|EPI_ISL_412898\|2019-12-30 |
| hCoV-19/Wuhan/HBCDC-HB-01/2019\|EPI_ISL_402132\|2019-12-30 |
| hCoV-19/Wuhan/IVDC-HB-envF13-20/2020\|EPI_ISL_408514\|2020-01-01 |
| hCoV-19/Wuhan/IPBCAMS-WH-05/2020\|EPI_ISL_403928\|2020-01-01 |
| hCoV-19/Wuhan/IPBCAMS-WH-03/2019\|EPI_ISL_403930\|2019-12-30 |
| hCoV-19/Wuhan/HBCDC-HB-03/2019\|EPI_ISL_412899\|2019-12-30 |
| hCoV-19/Wuhan/WIV06/2019\|EPI_ISL_402129\|2019-12-30 |
| hCoV-19/Wuhan/WHU02/2020\|EPI_ISL_406717\|2020-01-02 |
| hCoV-19/Wuhan/WHU01/2020\|EPI_ISL_406716\|2020-01-02 |
| hCoV-19/Wuhan/IPBCAMS-WH-04/2019\|EPI_ISL_403929\|2019-12-30 |
| hCoV-19/Wuhan/WH03/2020\|EPI_ISL_406800\|2020-01-01 |
| hCoV-19/Wuhan/IVDC-HB-01/2019\|EPI_ISL_402119\|2019-12-30 |
| hCoV-19/Wuhan/WIV04/2019\|EPI_ISL_402124\|2019-12-30 |
|  |
|  |
| **Italy** |
|  |
| hCoV-19/Italy/NIAA05032020/2020\|EPI_ISL_414512\|2020-03-05 |
| hCoV-19/Italy/TE5476/2020\|EPI_ISL_420565\|2020-03-19 |
| hCoV-19/Italy/INMI1/2020\|EPI_ISL_408068\|2020-01-29 |
| hCoV-19/Italy/INMI7/2020\|EPI_ISL_419255\|2020-03-23 |
| hCoV-19/Italy/INMI6/2020\|EPI_ISL_419254\|2020-03-23 |
| hCoV-19/Italy/MBS-Cagliari-1/2020\|EPI_ISL_414599\|2020-03 |
| hCoV-19/Italy/SPL1/2020\|EPI_ISL_412974\|2020-01-29 |
| hCoV-19/Italy/CDG1/2020\|EPI_ISL_412973\|2020-02-20 |
| hCoV-19/Italy/INMI4/2020\|EPI_ISL_417922\|2020-02-28 |
| hCoV-19/Italy/INMI3/2020\|EPI_ISL_417921\|2020-03-01 |
| hCoV-19/Italy/INMI5/2020\|EPI_ISL_417923\|2020-03-04 |
| hCoV-19/Italy/VR_20COV_21-37/2020\|EPI_ISL_422438\|2020-03-25 |
| hCoV-19/Italy/VR_20COV_21-26/2020\|EPI_ISL_422437\|2020-03-25 |
| hCoV-19/Italy/INMI1-isl/2020\|EPI_ISL_410545\|2020-01-29 |
| hCoV-19/Italy/INMI1-cs/2020\|EPI_ISL_410546\|2020-01-31 |
| hCoV-19/Italy/UniMI01/2020\|EPI_ISL_417445\|2020-02-24 |
| hCoV-19/Italy/UniMI03/2020\|EPI_ISL_417447\|2020-02-24 |
| hCoV-19/Italy/UniMI02/2020\|EPI_ISL_417446\|2020-02-24 |
| hCoV-19/Italy/FVG-ICGEB_S1/2020\|EPI_ISL_417418\|2020-03-01 |
| hCoV-19/Italy/FVG-ICGEB_S5/2020\|EPI_ISL_417419\|2020-03-01 |
| hCoV-19/Italy/FVG-ICGEB_S9/2020\|EPI_ISL_417423\|2020-03-01 |
| hCoV-19/Italy/FVG-ICGEB_S8/2020\|EPI_ISL_417421\|2020-03-01 |
| hCoV-19/Italy/UnivPM1/2020\|EPI_ISL_417491\|2020-03-03 |
| hCoV-19/Italy/UniSR1/2020\|EPI_ISL_413489\|2020-03-03 |
| hCoV-19/Italy/TE4836/2020\|EPI_ISL_418260\|2020-03-16 |
| hCoV-19/Italy/TE5052/2020\|EPI_ISL_418261\|2020-03-17 |
| hCoV-19/Italy/TE6222/2020\|EPI_ISL_420583\|2020-03-23 |
| hCoV-19/Italy/TE6225/2020\|EPI_ISL_420592\|2020-03-23 |
| hCoV-19/Italy/TE5166/2020\|EPI_ISL_420563\|2020-03-18 |
| hCoV-19/Italy/TE5472/2020\|EPI_ISL_420564\|2020-03-19 |
| hCoV-19/Italy/TE5780/2020\|EPI_ISL_420567\|2020-03-21 |
| hCoV-19/Italy/6193/2020\|EPI_ISL_420568\|2020-03-23 |
| hCoV-19/Italy/TE5512/2020\|EPI_ISL_420566\|2020-03-19 |
| hCoV-19/Italy/TE6195/2020\|EPI_ISL_420569\|2020-03-23 |
| hCoV-19/Italy/TE4953/2020\|EPI_ISL_418258\|2020-03-14 |
| hCoV-19/Italy/TE4959/2020\|EPI_ISL_418259\|2020-03-14 |
| hCoV-19/Italy/TE4925/2020\|EPI_ISL_418255\|2020-03-14 |
| hCoV-19/Italy/TE4880/2020\|EPI_ISL_418256\|2020-03-14 |
| hCoV-19/Italy/TE5056/2020\|EPI_ISL_418257\|2020-03-17 |
| hCoV-19/Italy/INMI8/2020\|EPI_ISL_424342\|2020-03-07 |
| hCoV-19/Italy/INMI9/2020\|EPI_ISL_424343\|2020-03-23 |
| hCoV-19/Italy/INMI10/2020\|EPI_ISL_424344\|2020-03-04 |
| hCoV-19/Italy/INMI1/2020\|EPI_ISL_406959\|2020-01 |
| hCoV-19/Italy/INMI2/2020\|EPI_ISL_406960\|2020-01 |
|  |
|  |
|  |
|  |
| **Spain** |
| hCoV-19/Spain/Valencia52/2020\|EPI_ISL_421519\|2020-03-21 |
| hCoV-19/Spain/Valencia40/2020\|EPI_ISL_421515\|2020-03-07 |
| hCoV-19/Spain/Valencia6/2020\|EPI_ISL_416485\|2020-02-27 |
| hCoV-19/Spain/Valencia19/2020\|EPI_ISL_419683\|2020-03-08 |
| hCoV-19/Spain/Valencia72/2020\|EPI_ISL_425196\|2020-03-17 |
| hCoV-19/Spain/Valencia18/2020\|EPI_ISL_419682\|2020-03-10 |
| hCoV-19/Spain/Valencia73/2020\|EPI_ISL_425197\|2020-03-17 |
| hCoV-19/Spain/Valencia69/2020\|EPI_ISL_425193\|2020-03-17 |
| hCoV-19/Spain/Madrid_H8_37/2020\|EPI_ISL_418182\|2020-03-12 |
| hCoV-19/Spain/Madrid_H7_36/2020\|EPI_ISL_417957\|2020-03-12 |
| hCoV-19/Spain/Madrid_H5_34/2020\|EPI_ISL_417956\|2020-03-11 |
| hCoV-19/Spain/Valencia56/2020\|EPI_ISL_425180\|2020-03-10 |
| hCoV-19/Spain/Valencia54/2020\|EPI_ISL_425178\|2020-02-28 |
| hCoV-19/Spain/Madrid_R2_15/2020\|EPI_ISL_417979\|2020-03-03 |
| hCoV-19/Spain/Valencia95/2020\|EPI_ISL_425219\|2020-03-17 |
| hCoV-19/Spain/PaisVasco201493/2020\|EPI_ISL_419238\|2020-03-05 |
| hCoV-19/Spain/LaRioja201575/2020\|EPI_ISL_419234\|2020-03-03 |
| hCoV-19/Spain/Canarias201495/2020\|EPI_ISL_419233\|2020-03-04 |
| hCoV-19/Spain/Valencia5/2020\|EPI_ISL_416484\|2020-02-27 |
| hCoV-19/Spain/Valencia15/2020\|EPI_ISL_419679\|2020-03-02 |
| hCoV-19/Spain/Valencia3/2020\|EPI_ISL_414598\|2020-03-05 |
| hCoV-19/Spain/Valencia30/2020\|EPI_ISL_420115\|2020-03-09 |
| hCoV-19/Spain/Madrid_R10_33/2020\|EPI_ISL_417981\|2020-03-02 |
| hCoV-19/Spain/Valencia14/2020\|EPI_ISL_419678\|2020-03-09 |
| hCoV-19/Spain/Valencia28/2020\|EPI_ISL_420113\|2020-03-09 |
| hCoV-19/Spain/Valencia59/2020\|EPI_ISL_425183\|2020-03-10 |
| hCoV-19/Spain/Valencia58/2020\|EPI_ISL_425182\|2020-03-10 |
| hCoV-19/Spain/Valencia29/2020\|EPI_ISL_420114\|2020-03-09 |
| hCoV-19/Spain/Valencia34/2020\|EPI_ISL_420119\|2020-03-09 |
| hCoV-19/Spain/Valencia12/2020\|EPI_ISL_419676\|2020-03-09 |
| hCoV-19/Spain/Valencia8/2020\|EPI_ISL_416487\|2020-03-04 |
| hCoV-19/Spain/Valencia62/2020\|EPI_ISL_425186\|2020-03-10 |
| hCoV-19/Spain/Valencia61/2020\|EPI_ISL_425185\|2020-03-10 |
| hCoV-19/Spain/Valencia60/2020\|EPI_ISL_425184\|2020-03-10 |
| hCoV-19/Spain/Valencia57/2020\|EPI_ISL_425181\|2020-03-10 |
| hCoV-19/Spain/Valencia55/2020\|EPI_ISL_425179\|2020-03-10 |
| hCoV-19/Spain/Valencia93/2020\|EPI_ISL_425217\|2020-03-17 |
| hCoV-19/Spain/Valencia89/2020\|EPI_ISL_425213\|2020-03-11 |
| hCoV-19/Spain/Valencia27/2020\|EPI_ISL_420112\|2020-03-09 |
| hCoV-19/Spain/Valencia37/2020\|EPI_ISL_420122\|2020-03-09 |
| hCoV-19/Spain/Valencia36/2020\|EPI_ISL_420121\|2020-03-09 |
| hCoV-19/Spain/Valencia39/2020\|EPI_ISL_420124\|2020-03-09 |
| hCoV-19/Spain/Valencia38/2020\|EPI_ISL_420123\|2020-03-09 |
| hCoV-19/Spain/Valencia35/2020\|EPI_ISL_420120\|2020-03-09 |
| hCoV-19/Spain/Valencia33/2020\|EPI_ISL_420118\|2020-03-09 |
| hCoV-19/Spain/Valencia32/2020\|EPI_ISL_420117\|2020-03-09 |
| hCoV-19/Spain/Valencia31/2020\|EPI_ISL_420116\|2020-03-09 |
| hCoV-19/Spain/Madrid_LP14_3/2020\|EPI_ISL_417975\|2020-03-09 |
| hCoV-19/Spain/Valencia11/2020\|EPI_ISL_419675\|2020-03-20 |
| hCoV-19/Spain/Madrid201738/2020\|EPI_ISL_419237\|2020-03-07 |
| hCoV-19/Spain/Madrid_R5_8/2020\|EPI_ISL_417980\|2020-03-03 |
| hCoV-19/Spain/Valencia92/2020\|EPI_ISL_425216\|2020-03-16 |
| hCoV-19/Spain/Valencia94/2020\|EPI_ISL_425218\|2020-03-17 |
| hCoV-19/Spain/Andalucia201617/2020\|EPI_ISL_419230\|2020-03-05 |
| hCoV-19/Spain/Madrid_H12_2309/2020\|EPI_ISL_421179\|2020-03-27 |
| hCoV-19/Spain/Valencia45/2020\|EPI_ISL_420129\|2020-03-13 |
| hCoV-19/Spain/VH198152683/2020\|EPI_ISL_418861\|2020-03-24 |
| hCoV-19/Spain/Valencia81/2020\|EPI_ISL_425205\|2020-03-20 |
| hCoV-19/Spain/Valencia79/2020\|EPI_ISL_425203\|2020-03-20 |
| hCoV-19/Spain/Madrid_H12_2208/2020\|EPI_ISL_421178\|2020-03-27 |
| hCoV-19/Spain/Valencia17/2020\|EPI_ISL_419681\|2020-03-10 |
| hCoV-19/Spain/Valencia41/2020\|EPI_ISL_420125\|2020-03-11 |
| hCoV-19/Spain/Madrid_H12_1905/2020\|EPI_ISL_421175\|2020-03-29 |
| hCoV-19/Spain/Valencia50/2020\|EPI_ISL_420131\|2020-03-18 |
| hCoV-19/Spain/Valencia97/2020\|EPI_ISL_425221\|2020-03-17 |
| hCoV-19/Spain/Valencia64/2020\|EPI_ISL_425188\|2020-03-16 |
| hCoV-19/Spain/Valencia77/2020\|EPI_ISL_425201\|2020-03-19 |
| hCoV-19/Spain/Valencia88/2020\|EPI_ISL_425212\|2020-03-11 |
| hCoV-19/Spain/Valencia86/2020\|EPI_ISL_425210\|2020-03-25 |
| hCoV-19/Spain/Valencia44/2020\|EPI_ISL_420128\|2020-03-12 |
| hCoV-19/Spain/Valencia65/2020\|EPI_ISL_425189\|2020-03-16 |
| hCoV-19/Spain/Madrid_H12_1502/2020\|EPI_ISL_421172\|2020-03-09 |
| hCoV-19/Spain/Madrid_H12_1804/2021\|EPI_ISL_421174\|2020-03-29 |
| hCoV-19/Spain/Madrid_H12_1703/2020\|EPI_ISL_421173\|2020-03-08 |
| hCoV-19/Spain/Madrid_H12_2107/2020\|EPI_ISL_421177\|2020-03-27 |
| hCoV-19/Spain/Madrid_H12_2410/2020\|EPI_ISL_421180\|2020-03-27 |
| hCoV-19/Spain/Valencia16/2020\|EPI_ISL_419680\|2020-03-10 |
| hCoV-19/Spain/Madrid_LP15_4/2020\|EPI_ISL_417978\|2020-03-09 |
| hCoV-19/Spain/Madrid_LP12_21/2020\|EPI_ISL_417972\|2020-03-09 |
| hCoV-19/Spain/Madrid_H11_40/2020\|EPI_ISL_417967\|2020-03-12 |
| hCoV-19/Spain/Madrid201706/2020\|EPI_ISL_419235\|2020-03-07 |
| hCoV-19/Spain/Valencia49/2020\|EPI_ISL_420130\|2020-03-18 |
| hCoV-19/Spain/Valencia51/2020\|EPI_ISL_420132\|2020-03-18 |
| hCoV-19/Spain/Valencia96/2020\|EPI_ISL_425220\|2020-03-17 |
| hCoV-19/Spain/Valencia75/2020\|EPI_ISL_425199\|2020-03-19 |
| hCoV-19/Spain/Valencia63/2020\|EPI_ISL_425187\|2020-03-16 |
| hCoV-19/Spain/Madrid_H12_2006/2020\|EPI_ISL_421176\|2020-03-27 |
| hCoV-19/Spain/Madrid_H12_1301/2020\|EPI_ISL_421171\|2020-03-05 |
| hCoV-19/Spain/Valencia42/2020\|EPI_ISL_420126\|2020-03-12 |
| hCoV-19/Spain/Madrid_H3_10/2020\|EPI_ISL_417954\|2020-03-12 |
| hCoV-19/Spain/Valencia13/2020\|EPI_ISL_419677\|2020-03-09 |
| hCoV-19/Spain/VH000001133/2020\|EPI_ISL_418860\|2020-03-15 |
| hCoV-19/Spain/Madrid_H10_39/2020\|EPI_ISL_417963\|2020-03-12 |
| hCoV-19/Spain/Valencia43/2020\|EPI_ISL_420127\|2020-03-12 |
| hCoV-19/Spain/Madrid201709/2020\|EPI_ISL_419236\|2020-03-07 |
| hCoV-19/Spain/Andalucia201272/2020\|EPI_ISL_418243\|2020-02-28 |
| hCoV-19/Spain/Cataluna201396/2020\|EPI_ISL_418250\|2020 |
| hCoV-19/Spain/Madrid201105/2020\|EPI_ISL_418251\|2020-02-25 |
| hCoV-19/Spain/Cataluna201397/2020\|EPI_ISL_419707\|2020 |
| hCoV-19/Spain/Madrid201442/2020\|EPI_ISL_417010\|2020-03-04 |
| hCoV-19/Spain/Galicia201663/2020\|EPI_ISL_417007\|2020-03-07 |
| hCoV-19/Spain/CastillayLeon201061/2020\|EPI_ISL_418247\|2020-02-26 |
| hCoV-19/Spain/PaisVasco201382/2020\|EPI_ISL_418253\|2020-03-02 |
| hCoV-19/Spain/PaisVasco201602/2020\|EPI_ISL_419709\|2020-03-04 |
| hCoV-19/Spain/CastillayLeon201372/2020\|EPI_ISL_418249\|2020-03-03 |
| hCoV-19/Spain/CastillayLeon201323/2020\|EPI_ISL_418248\|2020-03-01 |
| hCoV-19/Spain/PaisVasco201607/2020\|EPI_ISL_419240\|2020-03-03 |
| hCoV-19/Spain/CastillayLeon201437/2020\|EPI_ISL_416994\|2020-03-04 |
| hCoV-19/Spain/CastillaLaMancha201329/2020\|EPI_ISL_418246\|2020-03-01 |
| hCoV-19/Spain/CastillaLaMancha201328/2020\|EPI_ISL_418245\|2020-03-01 |
| hCoV-19/Spain/Valencia7/2020\|EPI_ISL_416486\|2020-03-02 |
| hCoV-19/Spain/Valencia90/2020\|EPI_ISL_425214\|2020-03-11 |
|  |
|  |
|  |
|  |
| **France** |
|  |
| hCoV-19/France/HF1805/2020\|EPI_ISL_414628\|2020-03-02 |
| hCoV-19/France/HF3293/2020\|EPI_ISL_421510\|2020-03-23 |
| hCoV-19/France/HF2174/2020\|EPI_ISL_415654\|2020-03-09 |
| hCoV-19/France/IDF3212/2020\|EPI_ISL_420061\|2020-03-23 |
| hCoV-19/France/IDF2075/2020\|EPI_ISL_415650\|2020-03-02 |
| hCoV-19/France/IDF2284/2020\|EPI_ISL_416501\|2020-03-10 |
| hCoV-19/France/HF3141/2020\|EPI_ISL_420057\|2020-03-22 |
| hCoV-19/France/HF1871/2020\|EPI_ISL_414630\|2020-03-03 |
| hCoV-19/France/HF2234/2020\|EPI_ISL_416495\|2020-03-10 |
| hCoV-19/France/HF3290/2020\|EPI_ISL_421509\|2020-03-23 |
| hCoV-19/France/HF2405/2020\|EPI_ISL_418228\|2020-03-12 |
| hCoV-19/France/HF2381/2020\|EPI_ISL_418226\|2020-03-09 |
| hCoV-19/France/HF1988/2020\|EPI_ISL_414635\|2020-03-04 |
| hCoV-19/France/HF1465/2020\|EPI_ISL_418218\|2020-02-21 |
| hCoV-19/France/IDF3274/2020\|EPI_ISL_421507\|2020-03-23 |
| hCoV-19/France/HF3295/2020\|EPI_ISL_421511\|2020-03-23 |
| hCoV-19/France/IDF3163/2020\|EPI_ISL_420058\|2020-03-20 |
| hCoV-19/France/IDF2256/2020\|EPI_ISL_416498\|2020-03-11 |
| hCoV-19/France/GE1977/2020\|EPI_ISL_414632\|2020-03-04 |
| hCoV-19/France/ARA10910/2020\|EPI_ISL_418430\|2020-03-18 |
| hCoV-19/France/IDF2768/2020\|EPI_ISL_420042\|2020-03-17 |
| hCoV-19/France/HF2239/2020\|EPI_ISL_416497\|2020-03-10 |
| hCoV-19/France/IDF2410/2020\|EPI_ISL_418229\|2020-03-12 |
| hCoV-19/France/ARA12388/2020\|EPI_ISL_420606\|2020-03-22 |
| hCoV-19/France/HF1995/2020\|EPI_ISL_414638\|2020-03-04 |
| hCoV-19/France/HF1993/2020\|EPI_ISL_414637\|2020-03-04 |
| hCoV-19/France/HF2393/2020\|EPI_ISL_418227\|2020-03-12 |
| hCoV-19/France/HF1684/2020\|EPI_ISL_414626\|2020-02-29 |
| hCoV-19/France/B2330/2020\|EPI_ISL_416502\|2020-02-26 |
| hCoV-19/France/HF2748/2020\|EPI_ISL_420041\|2020-03-17 |
| hCoV-19/France/IDF3170/2020\|EPI_ISL_420060\|2020-03-20 |
| hCoV-19/France/GE1583/2020\|EPI_ISL_414623\|2020-02-25 |
| hCoV-19/France/ARA10282/2020\|EPI_ISL_418428\|2020-03-17 |
| hCoV-19/France/HF2237/2020\|EPI_ISL_416496\|2020-03-10 |
| hCoV-19/France/IDF3345/2020\|EPI_ISL_421513\|2020-03-23 |
| hCoV-19/France/CVL3365/2020\|EPI_ISL_421514\|2020-03-20 |
| hCoV-19/France/PL1643/2020\|EPI_ISL_414625\|2020-02-26 |
| hCoV-19/France/ARA12485/2020\|EPI_ISL_420607\|2020-03-23 |
| hCoV-19/France/N1620/2020\|EPI_ISL_414624\|2020-02-26 |
| hCoV-19/France/ARA12524/2020\|EPI_ISL_420609\|2020-03-23 |
| hCoV-19/France/IDF3236/2020\|EPI_ISL_421506\|2020-03-21 |
| hCoV-19/France/N2223/2020\|EPI_ISL_416494\|2020-03-04 |
| hCoV-19/France/HF2496/2020\|EPI_ISL_418231\|2020-03-15 |
| hCoV-19/France/BFC2094/2020\|EPI_ISL_415651\|2020-03-05 |
| hCoV-19/France/BFC2147/2020\|EPI_ISL_415652\|2020-03-05 |
| hCoV-19/France/IDF3230/2020\|EPI_ISL_420063\|2020-03-22 |
| hCoV-19/France/BFC2709/2020\|EPI_ISL_420038\|2020-03-17 |
| hCoV-19/France/IDF2278/2020\|EPI_ISL_416499\|2020-03-11 |
| hCoV-19/France/ARA12576/2020\|EPI_ISL_420611\|2020-03-23 |
| hCoV-19/France/GE1973/2020\|EPI_ISL_414631\|2020-03-04 |
| hCoV-19/France/IDF3276/2020\|EPI_ISL_421508\|2020-03-23 |
| hCoV-19/France/IDF3165/2020\|EPI_ISL_420059\|2020-03-21 |
| hCoV-19/France/IDF3324/2020\|EPI_ISL_421512\|2020-03-23 |
| hCoV-19/France/GE2722/2020\|EPI_ISL_420040\|2020-03-12 |
| hCoV-19/France/IDF2420/2020\|EPI_ISL_418230\|2020-03-13 |
| hCoV-19/France/HF2196/2020\|EPI_ISL_416493\|2020-03-08 |
| hCoV-19/France/GE2720/2020\|EPI_ISL_420039\|2020-03-12 |
| hCoV-19/France/ARA10968/2020\|EPI_ISL_418431\|2020-03-18 |
| hCoV-19/France/ARA10184/2020\|EPI_ISL_418422\|2020-03-17 |
| hCoV-19/France/ARA10188/2020\|EPI_ISL_418423\|2020-03-17 |
| hCoV-19/France/ARA10876/2020\|EPI_ISL_418429\|2020-03-18 |
| hCoV-19/France/ARA10170/2020\|EPI_ISL_418420\|2020-03-17 |
| hCoV-19/France/Bourg-en-Bresse_06678/2020\|EPI_ISL_416757\|2020-03-07 |
| hCoV-19/France/ARA10189/2020\|EPI_ISL_418424\|2020-03-17 |
| hCoV-19/France/ARA12253/2020\|EPI_ISL_419183\|2020-03-22 |
| hCoV-19/France/ARA12270/2020\|EPI_ISL_419188\|2020-03-22 |
| hCoV-19/France/Clermont-Ferrand_650/2020\|EPI_ISL_416752\|2020-03-04 |
| hCoV-19/France/Lyon_06464 |
| hCoV-19/France/ARA10192/2020\|EPI_ISL_418425\|2020-03-17 |
| hCoV-19/France/ARA12250/2020\|EPI_ISL_419182\|2020-03-22 |
| hCoV-19/France/ARA09686/2020\|EPI_ISL_418417\|2020-03-16 |
| hCoV-19/France/Lyon_06531/2020\|EPI_ISL_416756\|2020-03-06 |
| hCoV-19/France/ARA12371/2020\|EPI_ISL_420604\|2020-03-23 |
| hCoV-19/France/HF3138/2020\|EPI_ISL_420056\|2020-03-22 |
| hCoV-19/France/IDF3235/2020\|EPI_ISL_420064\|2020-03-23 |
| hCoV-19/France/Bourg-en-Bresse_06813/2020\|EPI_ISL_417340\|2020-03-07 |
| hCoV-19/France/ARA12264/2020\|EPI_ISL_419185\|2020-03-22 |
| hCoV-19/France/IDF2548/2020\|EPI_ISL_421505\|2020-03-15 |
| hCoV-19/France/ARA12222/2020\|EPI_ISL_419179\|2020-03-22 |
| hCoV-19/France/IDF2414/2020\|EPI_ISL_421503\|2020-03-12 |
| hCoV-19/France/HF1645/2020\|EPI_ISL_418220\|2020-02-28 |
| hCoV-19/France/IDF2412/2020\|EPI_ISL_421502\|2020-03-12 |
| hCoV-19/France/ARA10251/2020\|EPI_ISL_418426\|2020-03-17 |
| hCoV-19/France/ARA12558/2020\|EPI_ISL_420610\|2020-03-23 |
| hCoV-19/France/ARA12384/2020\|EPI_ISL_420605\|2020-03-22 |
| hCoV-19/France/ARA10163/2020\|EPI_ISL_418418\|2020-03-16 |
| hCoV-19/France/ARA10257/2020\|EPI_ISL_418427\|2020-03-17 |
| hCoV-19/France/IDF2536/2020\|EPI_ISL_421504\|2020-03-14 |
| hCoV-19/France/ARA10165/2020\|EPI_ISL_418419\|2020-03-16 |
| hCoV-19/France/Lyon_06820/2020\|EPI_ISL_417339\|2020-03-08 |
| hCoV-19/France/Lyon_06487/2020\|EPI_ISL_416754\|2020-03-06 |
| hCoV-19/France/Lyon_0693/2020\|EPI_ISL_416758\|2020-03-08 |
| hCoV-19/France/ARA12269/2020\|EPI_ISL_419187\|2020-03-22 |
| hCoV-19/France/ARA12249/2020\|EPI_ISL_419181\|2020-03-22 |
| hCoV-19/France/ARA12265/2020\|EPI_ISL_419186\|2020-03-22 |
| hCoV-19/France/HF2306/2020\|EPI_ISL_421500\|2020-03-11 |
| hCoV-19/France/ARA09588/2020\|EPI_ISL_418416\|2020-03-16 |
| hCoV-19/France/ARA12260/2020\|EPI_ISL_419184\|2020-03-22 |
| hCoV-19/France/ARA12238/2020\|EPI_ISL_419180\|2020-03-22 |
| hCoV-19/France/CVL2000/2020\|EPI_ISL_418222\|2020-03-04 |
| hCoV-19/France/HF2060/2020\|EPI_ISL_418223\|2020-03-05 |
| hCoV-19/France/HF2155/2020\|EPI_ISL_418225\|2020-03-08 |
| hCoV-19/France/HF2150/2020\|EPI_ISL_418224\|2020-03-08 |
| hCoV-19/France/B1623/2020\|EPI_ISL_418219\|2020-02-26 |
|  |
|  |
| **England** |
|  |
|  |
| hCoV-19/England/20102068502/2020\|EPI_ISL_417213\|2020-03-01 |
| hCoV-19/England/201360299/2020\|EPI_ISL_421778\|2020-03-25 |
| hCoV-19/England/201360185/2020\|EPI_ISL_421771\|2020-03-24 |
| hCoV-19/England/20139048804/2020\|EPI_ISL_420698\|2020-03-28 |
| hCoV-19/England/20139037504/2020\|EPI_ISL_420670\|2020-03-27 |
| hCoV-19/England/20140038604/2020\|EPI_ISL_420750\|2020-03-23 |
| hCoV-19/England/20109039306/2020\|EPI_ISL_417262\|2020-03-06 |
| hCoV-19/England/20139063604/2020\|EPI_ISL_420746\|2020-03-25 |
| hCoV-19/England/20104008702/2020\|EPI_ISL_417230\|2020-03-02 |
| hCoV-19/England/20112112106/2020\|EPI_ISL_417314\|2020-03-10 |
| hCoV-19/England/20124001902/2020\|EPI_ISL_418686\|2020-03-17 |
| hCoV-19/England/20139047504/2020\|EPI_ISL_420694\|2020-03-28 |
| hCoV-19/England/201360289/2020\|EPI_ISL_421773\|2020-03-26 |
| hCoV-19/England/20126035502/2020\|EPI_ISL_418764\|2020-03-18 |
| hCoV-19/England/20128046202/2020\|EPI_ISL_420478\|2020-03-19 |
| hCoV-19/England/20124030003/2020\|EPI_ISL_418707\|2020-03-17 |
| hCoV-19/England/201360508/2020\|EPI_ISL_421783\|2020-03-25 |
| hCoV-19/England/20126007102/2020\|EPI_ISL_418751\|2020-03-18 |
| hCoV-19/England/20109053606/2020\|EPI_ISL_417279\|2020-03-07 |
| hCoV-19/England/20109050706/2020\|EPI_ISL_417268\|2020-03-05 |
| hCoV-19/England/20124020402/2020\|EPI_ISL_418701\|2020-03-17 |
| hCoV-19/England/201360297/2020\|EPI_ISL_421776\|2020-03-25 |
| hCoV-19/England/20124101102/2020\|EPI_ISL_418739\|2020-03-18 |
| hCoV-19/England/20104003002/2020\|EPI_ISL_417222\|2020-03-04 |
| hCoV-19/England/201360499/2020\|EPI_ISL_421781\|2020-03-25 |
| hCoV-19/England/20139057004/2020\|EPI_ISL_420717\|2020-03-26 |
| hCoV-19/England/20139063204/2020\|EPI_ISL_420744\|2020-03-28 |
| hCoV-19/England/201380069/2020\|EPI_ISL_421817\|2020-03-25 |
| hCoV-19/England/SHEF-BFDFA/2020\|EPI_ISL_418294\|2020-03-18 |
| hCoV-19/England/20139055304/2020\|EPI_ISL_420715\|2020-03-29 |
| hCoV-19/England/20140039004/2020\|EPI_ISL_420752\|2020-03-26 |
| hCoV-19/England/200981386/2020\|EPI_ISL_414010\|2020-02-26 |
| hCoV-19/England/20124008902/2020\|EPI_ISL_418694\|2020-03-16 |
| hCoV-19/England/20120033002/2020\|EPI_ISL_420464\|2020-03-12 |
| hCoV-19/England/20124002102/2020\|EPI_ISL_418688\|2020-03-17 |
| hCoV-19/England/20139063804/2020\|EPI_ISL_420748\|2020-03-28 |
| hCoV-19/England/SHEF-BFCEE/2020\|EPI_ISL_416733\|2020-03-07 |
| hCoV-19/England/SHEF-C0628/2020\|EPI_ISL_420237\|2020-03-23 |
| hCoV-19/England/SHEF-C0585/2020\|EPI_ISL_420247\|2020-03-25 |
| hCoV-19/England/SHEF-C05C1/2020\|EPI_ISL_420282\|2020-03-21 |
| hCoV-19/England/SHEF-C053A/2020\|EPI_ISL_420235\|2020-03-23 |
| hCoV-19/England/SHEF-C00B1/2020\|EPI_ISL_420276\|2020-03-21 |
| hCoV-19/England/SHEF-C0682/2020\|EPI_ISL_420232\|2020-03-25 |
| hCoV-19/England/SHEF-C0549/2020\|EPI_ISL_420249\|2020-03-26 |
| hCoV-19/England/SHEF-BFF6D/2020\|EPI_ISL_418313\|2020-03-24 |
| hCoV-19/England/SHEF-C034F/2020\|EPI_ISL_420205\|2020-03-29 |
| hCoV-19/England/SHEF-C0093/2020\|EPI_ISL_420268\|2020-03-23 |
| hCoV-19/England/SHEF-C0646/2020\|EPI_ISL_420278\|2020-03-21 |
| hCoV-19/England/SHEF-C05B2/2020\|EPI_ISL_420244\|2020-03-26 |
| hCoV-19/England/SHEF-C0084/2020\|EPI_ISL_420240\|2020-03-23 |
| hCoV-19/England/SHEF-C04D3/2020\|EPI_ISL_420213\|2020-03-29 |
| hCoV-19/England/SHEF-C0233/2020\|EPI_ISL_420199\|2020-03-28 |
| hCoV-19/England/SHEF-BFFB8/2020\|EPI_ISL_418318\|2020-03-25 |
| hCoV-19/England/SHEF-C0619/2020\|EPI_ISL_420289\|2020-03-17 |
| hCoV-19/England/SHEF-BFEE8/2020\|EPI_ISL_418306\|2020-03-25 |
| hCoV-19/England/SHEF-C079E/2020\|EPI_ISL_420157\|2020-03-28 |
| hCoV-19/England/SHEF-C0251/2020\|EPI_ISL_420196\|2020-03-28 |
| hCoV-19/England/SHEF-C0804/2020\|EPI_ISL_420167\|2020-03-25 |
| hCoV-19/England/SHEF-C04B5/2020\|EPI_ISL_420202\|2020-03-29 |
| hCoV-19/England/SHEF-C08B9/2020\|EPI_ISL_420171\|2020-03-24 |
| hCoV-19/England/SHEF-C08C8/2020\|EPI_ISL_420165\|2020-03-25 |
| hCoV-19/England/SHEF-C044C/2020\|EPI_ISL_420193\|2020-03-28 |
| hCoV-19/England/SHEF-C0312/2020\|EPI_ISL_420216\|2020-03-29 |
| hCoV-19/England/SHEF-C0707/2020\|EPI_ISL_420270\|2020-03-22 |
| hCoV-19/England/SHEF-C07BC/2020\|EPI_ISL_420159\|2020-03-29 |
| hCoV-19/England/SHEF-C041F/2020\|EPI_ISL_420210\|2020-03-27 |
| hCoV-19/England/SHEF-BFCC0/2020\|EPI_ISL_416731\|2020-03-03 |
| hCoV-19/England/SHEF-C0734/2020\|EPI_ISL_420242\|2020-03-26 |
| hCoV-19/England/200960041/2020\|EPI_ISL_414008\|2020-02-27 |
| hCoV-19/England/200990002/2020\|EPI_ISL_414522\|2020-02-28 |
| hCoV-19/England/20108034006/2020\|EPI_ISL_417258\|2020-03-06 |
| hCoV-19/England/20139062704/2020\|EPI_ISL_420742\|2020-03-28 |
| hCoV-19/England/20122119502/2020\|EPI_ISL_418681\|2020-03-17 |
| hCoV-19/England/201380054/2020\|EPI_ISL_421809\|2020-03-25 |
| hCoV-19/England/201380217/2020\|EPI_ISL_421822\|2020-03-25 |
| hCoV-19/England/201380042/2020\|EPI_ISL_421804\|2020-03-25 |
| hCoV-19/England/20104008902/2020\|EPI_ISL_417232\|2020-03-04 |
| hCoV-19/England/200990723/2020\|EPI_ISL_414012\|2020-02-27 |
| hCoV-19/England/20139060804/2020\|EPI_ISL_420734\|2020-03-27 |
| hCoV-19/England/20124021402/2020\|EPI_ISL_418704\|2020-03-17 |
| hCoV-19/England/201380063/2020\|EPI_ISL_421815\|2020-03-25 |
| hCoV-19/England/20109051806/2020\|EPI_ISL_417270\|2020-03-06 |
| hCoV-19/England/20110023706/2020\|EPI_ISL_417307\|2020-03-08 |
| hCoV-19/England/20139045904/2020\|EPI_ISL_420687\|2020-03-28 |
| hCoV-19/England/20109050506/2020\|EPI_ISL_417266\|2020-03-05 |
| hCoV-19/England/201380056/2020\|EPI_ISL_421811\|2020-03-25 |
| hCoV-19/England/20139046604/2020\|EPI_ISL_420689\|2020-03-28 |
| hCoV-19/England/201360017/2020\|EPI_ISL_421768\|2020-03-24 |
| hCoV-19/England/20139060404/2020\|EPI_ISL_420732\|2020-03-28 |
| hCoV-19/England/201361008/2020\|EPI_ISL_421785\|2020-03-25 |
| hCoV-19/England/200990724/2020\|EPI_ISL_414006\|2020-02-28 |
| hCoV-19/England/20124003902/2020\|EPI_ISL_418691\|2020-03-17 |
| hCoV-19/England/201380040/2020\|EPI_ISL_421802\|2020-03-27 |
| hCoV-19/England/20104007503/2020\|EPI_ISL_417227\|2020-03-03 |
| hCoV-19/England/01/2020\|EPI_ISL_407071\|2020-01-29 |
| hCoV-19/England/09c/2020\|EPI_ISL_412116\|2020-02-09 |
| hCoV-19/England/20108007002/2020\|EPI_ISL_417255\|2020-03-04 |
| hCoV-19/England/20109056906/2020\|EPI_ISL_417282\|2020-03-05 |
| hCoV-19/England/SHEF-BFEBB/2020\|EPI_ISL_418304\|2020-03-24 |
| hCoV-19/England/SHEF-BFE9D/2020\|EPI_ISL_418302\|2020-03-24 |
| hCoV-19/England/SHEF-BFD45/2020\|EPI_ISL_416739\|2020-03-09 |
| hCoV-19/England/Sheff01/2020\|EPI_ISL_414500\|2020-03-04 |
| hCoV-19/England/201380049/2020\|EPI_ISL_421806\|2020-03-25 |
| hCoV-19/England/20139047104/2020\|EPI_ISL_420692\|2020-03-29 |
|  |
|  |
| **USA** |
|  |
| hCoV-19/USA/WA13-UW9/2020\|EPI_ISL_413601\|2020-03-02 |
| hCoV-19/USA/UT-00008/2020\|EPI_ISL_417026\|2020-03-20 |
| hCoV-19/USA/WA-UW154/2020\|EPI_ISL_416692\|2020-03-14 |
| hCoV-19/USA/WA-UW182/2020\|EPI_ISL_416720\|2020-03-13 |
| hCoV-19/USA/WA-UW49/2020\|EPI_ISL_415614\|2020-03-09 |
| hCoV-19/USA/WA-UW48/2020\|EPI_ISL_415613\|2020-03-09 |
| hCoV-19/USA/WA-UW94/2020\|EPI_ISL_416450\|2020-03-11 |
| hCoV-19/USA/WA-UW120/2020\|EPI_ISL_416658\|2020-03-11 |
| hCoV-19/USA/CA-CDPH-UC1/2020\|EPI_ISL_413557\|2020-02-28 |
| hCoV-19/USA/CA-CDPH-UC3/2020\|EPI_ISL_413559\|2020-02-27 |
| hCoV-19/USA/WI-02/2020\|EPI_ISL_416489\|2020-03-15 |
| hCoV-19/USA/WI-05/2020\|EPI_ISL_416492\|2020-03-15 |
| hCoV-19/USA/NY-NYUMC4/2020\|EPI_ISL_416832\|2020-03-16 |
| hCoV-19/USA/WA-UW96/2020\|EPI_ISL_416452\|2020-03-10 |
| hCoV-19/USA/WA-UW78/2020\|EPI_ISL_416434\|2020-03-10 |
| hCoV-19/USA/WA-UW110/2020\|EPI_ISL_416648\|2020-03-11 |
| hCoV-19/USA/WA-UW187/2020\|EPI_ISL_416725\|2020-03-13 |
| hCoV-19/USA/UPHL-05/2020\|EPI_ISL_415543\|2020-03-13 |
| hCoV-19/USA/WA-UW122/2020\|EPI_ISL_416660\|2020-03-10 |
| hCoV-19/USA/NY-NYUMC2/2020\|EPI_ISL_416830\|2020-03-16 |
| hCoV-19/USA/CruiseA-24/2020\|EPI_ISL_414483\|2020-02-17 |
| hCoV-19/USA/CruiseA-26/2020\|EPI_ISL_414485\|2020-02-24 |
| hCoV-19/USA/CA4/2020\|EPI_ISL_408009\|2020-01-29 |
| hCoV-19/USA/CruiseA-4/2020\|EPI_ISL_413609\|2020-02-21 |
| hCoV-19/USA/CruiseA-2/2020\|EPI_ISL_413607\|2020-02-18 |
| hCoV-19/USA/CruiseA-6/2020\|EPI_ISL_413611\|2020-02-21 |
| hCoV-19/USA/CruiseA-10/2020\|EPI_ISL_413615\|2020-02-17 |
| hCoV-19/USA/CruiseA-8/2020\|EPI_ISL_413613\|2020-02-17 |
| hCoV-19/USA/CruiseA-19/2020\|EPI_ISL_414479\|2020-02-18 |
| hCoV-19/USA/CruiseA-22/2020\|EPI_ISL_414481\|2020-02-21 |
| hCoV-19/USA/IL2/2020\|EPI_ISL_410045\|2020-01-28 |
| hCoV-19/USA/CA1/2020\|EPI_ISL_406034\|2020-01-23 |
| hCoV-19/USA/NY2-PV08100/2020\|EPI_ISL_415151\|2020-03-04 |
| hCoV-19/USA/TX1/2020\|EPI_ISL_411956\|2020-02-11 |
| hCoV-19/USA/CT-UW158/2020\|EPI_ISL_416696\|2020-03-13 |
| hCoV-19/USA/CA7/2020\|EPI_ISL_411954\|2020-02-06 |
| hCoV-19/USA/AZ1/2020\|EPI_ISL_406223\|2020-01-22 |
| hCoV-19/USA/WA1/2020\|EPI_ISL_404895\|2020-01-19 |
| hCoV-19/USA/WA1-F6/2020\|EPI_ISL_407215\|2020-01-25 |
| hCoV-19/USA/WA-UW184/2020\|EPI_ISL_416722\|2020-03-12 |
| hCoV-19/USA/WA-S56/2020\|EPI_ISL_417109\|2020-03-05 |
| hCoV-19/USA/WA-UW72/2020\|EPI_ISL_415600\|2020-03-09 |
| hCoV-19/USA/WA-S58/2020\|EPI_ISL_417111\|2020-03-05 |
| hCoV-19/USA/WA-S7/2020\|EPI_ISL_416462\|2020-02-24 |
| hCoV-19/USA/WA-UW36/2020\|EPI_ISL_416453\|2020-03-07 |
| hCoV-19/USA/WA-UW38/2020\|EPI_ISL_416455\|2020-03-07 |
| hCoV-19/USA/WA-UW51/2020\|EPI_ISL_415616\|2020-03-08 |
| hCoV-19/USA/MN3-MDH3/2020\|EPI_ISL_414590\|2020-03-09 |
| hCoV-19/USA/WA-UW143/2020\|EPI_ISL_416681\|2020-03-11 |
| hCoV-19/USA/WA-UW112/2020\|EPI_ISL_416650\|2020-03-10 |
| hCoV-19/USA/WA-UW139/2020\|EPI_ISL_416677\|2020-03-12 |
| hCoV-19/USA/WA-S11/2020\|EPI_ISL_416466\|2020-03-03 |
| hCoV-19/USA/WA-S23/2020\|EPI_ISL_417076\|2020-03-02 |
| hCoV-19/USA/WA-UW114/2020\|EPI_ISL_416652\|2020-03-11 |
| hCoV-19/USA/WA-UW62/2020\|EPI_ISL_415627\|2020-03-09 |
| hCoV-19/USA/WA-UW57/2020\|EPI_ISL_415622\|2020-03-09 |
| hCoV-19/USA/WA-UW74/2020\|EPI_ISL_415602\|2020-03-10 |
| hCoV-19/USA/WA-UW149/2020\|EPI_ISL_416687\|2020-03-14 |
| hCoV-19/USA/WA-S107/2020\|EPI_ISL_417160\|2020-02-29 |
| hCoV-19/USA/WA-UW141/2020\|EPI_ISL_416679\|2020-03-11 |
| hCoV-19/USA/WA-UW145/2020\|EPI_ISL_416683\|2020-03-15 |
| hCoV-19/USA/WA-S83/2020\|EPI_ISL_417136\|2020-03-05 |
| hCoV-19/USA/WA-UW176/2020\|EPI_ISL_416714\|2020-03-14 |
| hCoV-19/USA/WA-UW43/2020\|EPI_ISL_415608\|2020-03-08 |
| hCoV-19/USA/WA-UW55/2020\|EPI_ISL_415620\|2020-03-09 |
| hCoV-19/USA/WA-S76/2020\|EPI_ISL_417129\|2020-03-05 |
| hCoV-19/USA/WA-S50/2020\|EPI_ISL_417103\|2020-03-05 |
| hCoV-19/USA/WA-S81/2020\|EPI_ISL_417134\|2020-02-26 |
| hCoV-19/USA/WA-S54/2020\|EPI_ISL_417107\|2020-03-05 |
| hCoV-19/USA/WA-S114/2020\|EPI_ISL_417167\|2020-03-05 |
| hCoV-19/USA/WA-S86/2020\|EPI_ISL_417139\|2020-03-01 |
| hCoV-19/USA/WA-S19/2020\|EPI_ISL_417072\|2020-03-02 |
| hCoV-19/USA/WA-S111/2020\|EPI_ISL_417164\|2020-03-07 |
| hCoV-19/USA/WA-S12/2020\|EPI_ISL_417065\|2020-03-03 |
| hCoV-19/USA/WA-UW76/2020\|EPI_ISL_415604\|2020-03-10 |
| hCoV-19/USA/WA-UW116/2020\|EPI_ISL_416654\|2020-03-11 |
| hCoV-19/USA/WA-UW20/2020\|EPI_ISL_414368\|2020-03-05 |
| hCoV-19/USA/WA-UW18/2020\|EPI_ISL_414366\|2020-03-05 |
| hCoV-19/USA/WA-UW23/2020\|EPI_ISL_414592\|2020-03-06 |
| hCoV-19/USA/WA-UW118/2020\|EPI_ISL_416656\|2020-03-11 |
| hCoV-19/USA/WA-UW92/2020\|EPI_ISL_416448\|2020-03-11 |
| hCoV-19/USA/UPHL-03/2020\|EPI_ISL_415541\|2020-03-13 |
| hCoV-19/USA/WA-S17/2020\|EPI_ISL_417070\|2020-03-03 |
| hCoV-19/USA/WA-S79/2020\|EPI_ISL_417132\|2020-03-05 |
| hCoV-19/USA/WA-S109/2020\|EPI_ISL_417162\|2020-03-01 |
| hCoV-19/USA/WA-S46/2020\|EPI_ISL_417099\|2020-02-29 |
| hCoV-19/USA/WA-S21/2020\|EPI_ISL_417074\|2020-03-02 |
| hCoV-19/USA/WA-S74/2020\|EPI_ISL_417127\|2020-03-05 |
| hCoV-19/USA/WA-S48/2020\|EPI_ISL_417101\|2020-02-29 |
| hCoV-19/USA/WA-S44/2020\|EPI_ISL_417097\|2020-02-28 |
| hCoV-19/USA/WA-S5/2020\|EPI_ISL_416460\|2020-02-29 |
| hCoV-19/USA/WA-UW124/2020\|EPI_ISL_416662\|2020-03-12 |
| hCoV-19/USA/WA-UW152/2020\|EPI_ISL_416690\|2020-03-13 |
| hCoV-19/USA/WA-S26/2020\|EPI_ISL_417079\|2020-03-02 |
| hCoV-19/USA/WA-S72/2020\|EPI_ISL_417125\|2020-03-06 |
| hCoV-19/USA/WA-S116/2020\|EPI_ISL_417169\|2020-03-02 |
| hCoV-19/USA/WA-S52/2020\|EPI_ISL_417105\|2020-03-03 |
| hCoV-19/USA/WA-S88/2020\|EPI_ISL_417141\|2020-03-01 |
| hCoV-19/USA/WA-S101/2020\|EPI_ISL_417154\|2020-02-28 |
| hCoV-19/USA/WA-S15/2020\|EPI_ISL_417068\|2020-03-02 |
| hCoV-19/USA/WA-UW147/2020\|EPI_ISL_416685\|2020-03-15 |
| hCoV-19/USA/WA-S105/2020\|EPI_ISL_417158\|2020-02-28 |
| hCoV-19/USA/WA-S103/2020\|EPI_ISL_417156\|2020-02-28 |
| hCoV-19/USA/WA-UW190/2020\|EPI_ISL_416728\|2020-03-13 |
|  |
|  |
| **India** |
| hCoV-19/India/1093/2020\|EPI_ISL_421663\|2020-03-10 |
| hCoV-19/India/1073/2020\|EPI_ISL_421662\|2020-03-10 |
| hCoV-19/India/1104/2020\|EPI_ISL_421665\|2020-03-10 |
| hCoV-19/India/1111/2020\|EPI_ISL_421666\|2020-03-10 |
| hCoV-19/India/1100/2020\|EPI_ISL_421664\|2020-03-10 |
| hCoV-19/India/1616/2020\|EPI_ISL_421669\|2020-03-12 |
| hCoV-19/India/1621/2020\|EPI_ISL_421671\|2020-03-12 |
| hCoV-19/India/1652/2020\|EPI_ISL_424363\|2020-03-12 |
| hCoV-19/India/1644/2020\|EPI_ISL_421672\|2020-03-12 |
| hCoV-19/India/1617/2020\|EPI_ISL_421670\|2020-03-12 |
| hCoV-19/India/1063/2020\|EPI_ISL_424361\|2020-03-10 |
| hCoV-19/India/1115/2020\|EPI_ISL_421667\|2020-03-10 |
| hCoV-19/India/1-31/2020\|EPI_ISL_413523\|2020-01-31 |
| hCoV-19/India/1135/2020\|EPI_ISL_424362\|2020-03-10 |
| hCoV-19/India/c32/2020\|EPI_ISL_420555\|2020-03-03 |
| hCoV-19/India/2020c32/2020\|EPI_ISL_420556\|2020 |
| hCoV-19/India/c31/2020\|EPI_ISL_426179\|2020-03-02 |
| hCoV-19/India/3239/2020\|EPI_ISL_424365\|2020-03-17 |
| hCoV-19/India/3118/2020\|EPI_ISL_424364\|2020-03-17 |
| hCoV-19/India/763/2020\|EPI_ISL_420543\|2020-03-03 |
| hCoV-19/India/770/2020\|EPI_ISL_420545\|2020-03-03 |
| hCoV-19/USA/IN_2001/2020\|EPI_ISL_424866\|2020-03-09 |
| hCoV-19/USA/IN-Lilly-IPB0170-4bc20/2020\|EPI_ISL_421279\|2020-03-19 |
| hCoV-19/USA/IN-Lilly-IPB0170-4bc21/2020\|EPI_ISL_421281\|2020-03-19 |
| hCoV-19/India/772/2020\|EPI_ISL_420547\|2020-03-03 |
| hCoV-19/India/2020781/2020\|EPI_ISL_420554\|2020 |
| hCoV-19/India/2020770/2020\|EPI_ISL_420546\|2020 |
| hCoV-19/India/781/2020\|EPI_ISL_420553\|2020-03-03 |
| hCoV-19/India/777/2020\|EPI_ISL_420551\|2020-03-03 |
| hCoV-19/India/2020773/2020\|EPI_ISL_420550\|2020 |
| hCoV-19/India/2020777/2020\|EPI_ISL_420552\|2020 |
| hCoV-19/India/2020772/2020\|EPI_ISL_420548\|2020 |
| hCoV-19/India/773/2020\|EPI_ISL_420549\|2020-03-03 |
| hCoV-19/India/2020763/2020\|EPI_ISL_420544\|2020 |
